# Association between diabetes mellitus and primary restenosis following endovascular treatment: a comprehensive meta-analysis of randomized controlled trials

**DOI:** 10.1186/s12933-024-02201-6

**Published:** 2024-04-22

**Authors:** Xiaolei Sun, Cheng Zhang, Yarong Ma, Yanzheng He, Xiaodong Zhang, Jianbo Wu

**Affiliations:** 1 Department of General Surgery (Vascular Surgery), Affiliated Hospital of Southwest Medical University, Luzhou, 646000 China; 2 Department of Interventional Medicine, Affiliated Hospital of Southwest Medical University, Luzhou, 646000 China; 3 Department of Pharmacology, Basic Medicine Research Innovation Center for Cardiometabolic Diseases, Ministry of Education, and Laboratory for Cardiovascular Pharmacology, School of Pharmacy, Southwest Medical University, Luzhou, 646000 China; 4 Laboratory of Nucleic Acids in Medicine for National High-Level Talents, Nucleic Acid Medicine of Luzhou Key Laboratory, Southwest Medical University, Luzhou, 646000 China; 5 Key Laboratory of Medical Electrophysiology, Ministry of Education and Medical Electrophysiological Key Laboratory of Sichuan Province, Collaborative Innovation Center for Prevention and Treatment of Cardiovascular Disease of Sichuan Province, Institute of Cardiovascular Research, Southwest Medical University, Luzhou, 646000 China; 6 Cardiovascular and Metabolic Diseases Key Laboratory of Luzhou, Luzhou, 646000 China; 7 Department of Ophthalmology, Affiliated Hospital of Southwest Medical University, Luzhou, 646000 China; 8 Chongqing Clinical Research Center for Reproductive Medicine, Center for Reproductive Medicine, Women and Children’s Hospital of Chongqing Medical University, Chongqing, China; 9 School of Cardiovascular Medicine and Sciences, Faculty of Life Science and Medicine, King’s College London British Heart Foundation Centre of Research Excellence, King’s College London, London, SE5 9NU UK; 10 Department of General Surgery, Center of Vascular and Interventional Surgery, The Third People’s Hospital of Chengdu, The Affiliated Hospital of Southwest Jiaotong University &The Second Affiliated Hospital of Chengdu, Chongqing Medical University, Chengdu, 610031 China

## Abstract

**Importance:**

Diabetes mellitus (DM) is thought to be closely related to arterial stenotic or occlusive disease caused by atherosclerosis. However, there is still no definitive clinical evidence to confirm that patients with diabetes have a higher risk of restenosis.

**Objective:**

This meta-analysis was conducted to determine the effect of DM on restenosis among patients undergoing endovascular treatment, such as percutaneous transluminal angioplasty (PTA) or stenting.

**Data sources and study selection:**

The PubMed/Medline, EMBASE and Cochrane Library electronic databases were searched from 01/1990 to 12/2022, without language restrictions. Trials were included if they satisfied the following eligibility criteria: (1) RCTs of patients with or without DM; (2) lesions confined to the coronary arteries or femoral popliteal artery; (3) endovascular treatment via PTA or stenting; and (4) an outcome of restenosis at the target lesion site. The exclusion criteria included the following: (1) greater than 20% of patients lost to follow-up and (2) a secondary restenosis operation.

**Data extraction and synthesis:**

Two researchers independently screened the titles and abstracts for relevance, obtained full texts of potentially eligible studies, and assessed suitability based on inclusion and exclusion criteria.. Disagreements were resolved through consultation with a third researcher. Treatment effects were measured by relative ratios (RRs) with 95% confidence intervals (CIs) using random effects models. The quality of the evidence was assessed using the Grading of Recommendations Assessment, Development and Evaluation (GRADE) criteria.

**Main outcomes and measures:**

The main observation endpoint was restenosis, including > 50% stenosis at angiography, or TLR of the primary operation lesion during the follow-up period.

**Results:**

A total of 31,066 patients from 20 RCTs were included. Patients with DM had a higher risk of primary restenosis after endovascular treatment (RR = 1.43, 95% CI: 1.25–1.62; p = 0.001).

**Conclusions and relevance:**

This meta-analysis of all currently available RCTs showed that patients with DM are more prone to primary restenosis after endovascular treatment.

Cardiovascular disease (CVD) has the highest mortality rate worldwide [1], and vascular stenotic and occlusive lesions are the main pathological cause of death and disability for CVD patients [2]. At present, the development of intravascular therapy technology provides a reliable method for the treatment of vascular occlusive diseases, and quite a few of the difficulties in treatment have been resolved. However, restenosis after endovascular repair is a major problem that confuses clinicians and affects their choice of treatment [3]. Studies have shown that 30% to 50% of patients with coronary ischemic disease experience restenosis after endovascular therapy within five years; although the application of drug-coated balloons or drug-eluting stents has clearly reduced the occurrence of restenosis, restenosis still occurs in 10% to 20% of patients within one year [4, 5]. Restenosis after endovascular treatment causes a large number of patients to stop working or even die, which causes considerable damage to people's health and social and economic development. Thus, this is an urgent unsolved clinical problem. Identifying potential exposure and protective factors could make health care more effective in controlling restenosis after endovascular treatment.

Diabetes mellitus (DM) and its complications constitute one of the greatest human health problems worldwide. The prevalence of DM will increase globally from 371 million individuals in 2013 to 552 million individuals in 2030 [6]. DM is an important risk factor for the development of atherosclerotic diseases such as coronary heart disease (CHD), cerebrovascular disease, and peripheral artery disease (PAD). Cardiovascular complications are the leading cause of mortality among individuals with DM, and > 50% of patients die from a cardiovascular event, especially coronary artery disease but also stroke and peripheral vascular disease [7]. Insulin resistance and hyperglycemia in diabetes patients increase the risk of adverse cardiovascular events [8]. Studies have shown that hyperglycemia, insulin resistance, and an increase in advanced glycation end products are important conditions for a 2–fourfold increased risk of coronary artery disease (CAD) and PAD among people with diabetes [9–14]. However, at present, there is insufficient clinical evidence to confirm that diabetes increases the risk of restenosis after endovascular treatment. Therefore, to determine the effect of DM on restenosis among patients following endovascular interventional therapy, we designed this meta-analysis based on current clinical randomized controlled trial (RCT) data.

## Methods

### Study design and search strategy

We have designed the research and register for the study on the INPLASY website, and the registration number is INPLASY202370034 (DOI: 10.37766 / inplasy2023.7.0034). Ethical approval was acquired from the Ethic Committee of Southwest Medical University (No. 20220217–013). Two researchers independently scanned the titles and abstracts of the retrieved studies for the topic, and then obtained the full texts of potentially eligible studies and examined these independently for their suitability according to the inclusion criteria. In the case of disagreement between the two researchers, a third researcher was consulted to reach a consensus on whether to include the report or not. They documented the selection process with a Preferred Reporting Items for Systematic Reviews and Meta-Analyses (PRISMA [15]) flow chart. Trials were included if they satisfied the following eligibility criteria: (1) The studies included had to be randomized controlled studies (RCTs) including patients with or without DM; (2) had to involve lesions confined to the coronary arteries or femoral popliteal artery; (3) had to involve endovascular treatment via percutaneous transluminal angioplasty (PTA) or stenting; and (4) had to include an outcome of restenosis at the target lesion site. The exclusion criteria were as follows: (1) the proportion of patients lost to follow-up was higher than 20%; (2) a secondary restenosis operation; and (3) a sub-analysis or post-hoc analysis of RCTs. We developed and adhered to a standard protocol for study identification, inclusion, and data abstraction for all steps of our systematic review. Our major endpoint was restenosis, defined as a stenosis diameter > 50% in the in-segment area assessed by angiography, including the stent area as well as 5-mm margins proximal and distal to the stent. Meanwhile, clinically driven target lesion revascularization (CD-TLR) was also included, defined as any procedure performed to restore luminal patency after there has been late luminal loss of the target lesion (confirmed by angiography).

In this meta-analysis, we identified related published studies through a computerized literature search of the PubMed/Medline, Cochrane Library, and EMBASE (from 01/1990 to 12/2022) electronic databases. Two independent researchers checked citations for inclusion in the systematic review by using a hierarchical approach. The researchers assessed the title, abstract, and full text of these manuscripts. In addition, another reviewer manually searched the bibliographies of journal articles and relevant reviews to locate additional studies.

Reviewers extracted data using a data extraction form designed and piloted by the authors. If studies were reported in multiple publications, data were extracted from the different publications and then combined into a single data extraction form so that no data were omitted. The following characteristics of the included studies were registered in the data extraction form: methods and study design, participants, interventions and outcomes, including the outcome of restenosis. For all unclear restenosis analysis results, emails were sent to the corresponding authors to request the raw data; unfortunately, up to the time that the manuscript was written, no reply had been received.

### Data synthesis and statistical analysis

For dichotomous data, we calculated Mantel‒Haenszel relative ratios (RRs) and 95% confidence intervals (CIs). Heterogeneity across studies was assessed by Cochran’s Q statistic with a P value set at 0.1. The I^2^ statistic was also taken into account regardless of the P value. An I^2^ of ≥ 50% was prespecified as the threshold considered too high to provide consistent analysis. A random effects model was used for the analysis. Tests were two-tailed, and a P value of < 0.05 was considered statistically significant. Funnel plots were used to assess publication bias. STATA 12.0 (StataCorp, USA) was used to analyze the data.

### Assessment of the risk of bias in the included studies

Two reviewers assessed the risk of bias independently for the included studies using the Cochrane risk of bias assessment tool [16]. We evaluated all included studies for the following: adequacy of sequence generation and allocation concealment; adequacy of blinding of couples, providers and outcome assessors; completeness of outcome data; risk of selective outcome reporting; and risk of other potential sources of bias. The results of the risk of bias assessment are presented in Fig. 2.

## Results

### The results of the literature search

**Fig. 1:**
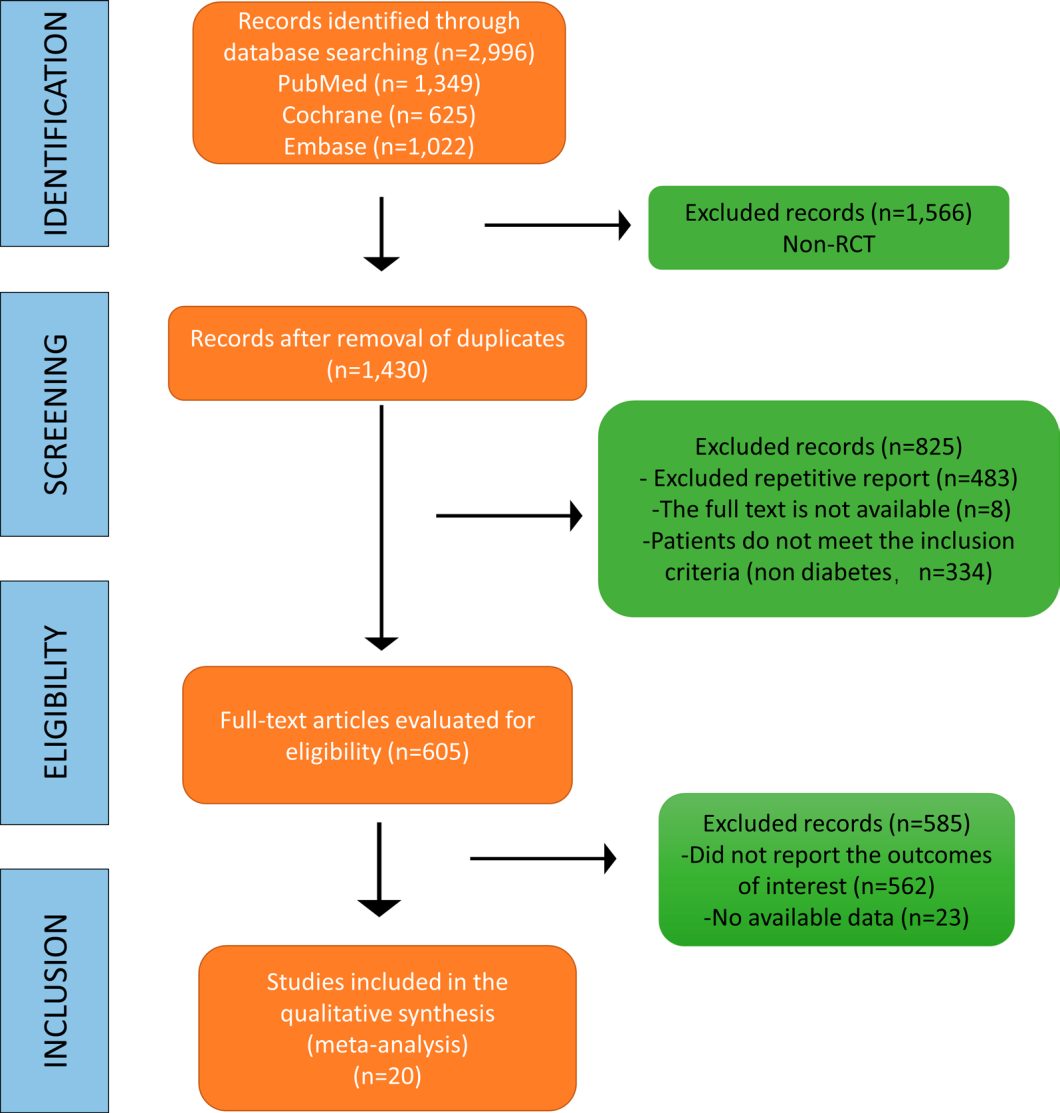
Flow diagram of the study selection process

In this analysis, a total of 2,996 RCTs were retrieved from the database. After eliminating duplicate studies, 1,430 studies remained. After browsing titles and abstracts, 605 full-text articles were assessed for eligibility, of which 585 were excluded because of the absence of relevant endpoint data (no surveillance undertaken, restenosis rates not reported separately). Finally, 20 studies were included in the meta-analysis for qualitative and quantitative analyses (Fig. 1). As a result, 31,066 patients were enrolled in this study. Table 1 details the case numbers and baseline characteristics, inclusion/exclusion criteria, number and type of stent/PTA procedure, strategies for follow-up, criteria for diagnosing restenosis, and the number of cases of restenosis with DM for each RCT. All authors of the studies included in this meta-analysis were requested to supply missing data and details of their studies. Unfortunately, no author supplied the requested information.

**Table 1:** Baseline characteristics and quality assessment of the included studies

Study and year	Inclusion criteria	Exclusion criteria	Baseline characteristics		Diabetic	Nondiabetic	Drugs before or during the procedure	Drugs after the procedure
AIDA [55] 2020	Consecutive patients from the general all-comer interventional cardiology population with 1 or more target lesions suitable for DES implantation according to the applicable local and European guidelines and the IFU of the AbsorbBVS are candidates for enrollment	1. Visually estimated target lesions more than 70 mm in length, 2. A reference vessel diameter visually estimated of < 2.5 mm or more than 4.0 mm, 3. Bifurcation lesions for which the use of two devices were needed, and 4. in-stent restenosis	Demographics	n	324	1521	Not available	The protocol mandates the prescription of dual-antiplatelet therapy (75–100 mg aspirin daily and 75 mg clopidogrel or 10 mg prasugrel or 180 mg ticagrelor daily) for a minimum of 1 year in the index and control strategies
				Age	66.28	64.26		
				Male sex (74.25%)	218 (67.28%)	1152 (75.74%)		
				Restenosis	31	92		
			Treatment	Oral medication	196 (60.49%)	-		
				Insulin	110 (33.95%)			
				None	18 (5.56%)			
				Unknown	4 (1.23%)			
			Risk factors	Hypertension (45.1%)	130 (40.12%)	702 (46.15%)		
				Hyperlipemia (37.6%)	176 (54.32%)	518 (34.06%)		
				Family history of CAD	146 (45.06%)	774 (50.89%)		
				Current smoker (28.2%)	66 (20.37%)	455 (29.91%)		
			Study design	Randomization	RCT			
				Blinding	Single-blind			
				Center	Multicenter, Europe			
				Endpoint of interest	TLR			
				Comparison	ABSORB everolimus-eluting bioresorbable vascular scaffold vs. the XIENCE family everolimus-eluting coronary stent system			
				Follow-up	3 years			
				Artery	Coronary			
BELLO [56] 2016	Eligible patients were age 18 years or older, with a diagnosis of stable or unstable angina or documented silent ischemia and a maximum of 2 angiographically significant de novo target lesions < 25 mm in length in native coronary arteries with a visually estimated RVD < 2.8 mm	Clinical exclusion criteria included acute myocardial infarction (MI) within the previous 48 h; previous percutaneous coronary intervention within the last 3 months; elective surgery planned within 6 months after the procedure; left ventricular ejection fraction < 30%; serum creatinine ≧2.0 μmol/l; contraindication or suspected intolerance to paclitaxel, aspirin, thienopyridines, or iodinated contrast that cannot be pretreated; platelet count < 50,000 cells/mm; positive pregnancy test; and stroke within the previous 6 months	Demographics	N	74	108	All patients were pretreated with aspirin and either ticlopidine or clopidogrel, anticoagulation was achieved with either intravenous unfractionated heparin or bivalirudin per standard of care	The protocol recommended that patients receive aspirin indefinitely and daily clopidogrel for a minimum of 1) 30 days in case of treatment with only DEB; 2) 3 months in case of provisional BMS after DEB; and 3) 12 months after DES implantation
				Age	68	64		
				Male sex (78.02%)	60	82		
				Restenosis	7	6		
			Treatment	Insulin	25 (33.78%)	-		
			Risk factors	Hypertension (80.2%)	65 (87.8%)	81 (75%)		
				Hyperlipemia (78.6%)	58 (78.38%)	85 (78.7%)		
				Family history of CAD	11 (14.86%)	35 (32.41%)		
				Current smoker (45.1%)	31 (41.89%)	51 (46.3%)		
			Study design	Randomization	RCT			
				Blinding	Single-blind			
				Center	Multicenter, Europe			
				Endpoint of interest	QA-R			
				Comparison	Drug-eluting balloons vs. paclitaxel-eluting stents			
				Follow-up	3 years			
				Artery	Coronary			
BIONICS [57] 2018	Patients with ischemic heart disease undergoing planned stent implantation were eligible for enrollment. Angiographic inclusion criteria included a reference vessel diameter between 2.5 mm and 4.25 mm, with a maximum of 2 lesions per vessel in up to 2 major coronary arteries	Patients with recent (< 24 h) ST-segment elevation MI, left ventricular ejection fraction < 30%, active stent thrombosis (ST), creatinine clearance < 30 ml/min, prior PCI within 12 months, and those unlikely to adhere to dual antiplatelet therapy were excluded	Demographics	N	559	1360	Before PCI, all patients received treatment with aspirin (325 mg if no prior therapy, 75–325 mg if chronic therapy) and either clopidogrel, ticagrelor or prasugrel per investigator discretion	Dual antiplatelet therapy use was mandatory for a minimum of 6 months following the procedure
				Age	64.0	63.2		
				Male sex (80.6%)	437 (78.2%)	1110 (80.9%)		
				Restenosis	38	46		
			Treatment	Not available				
			Risk factors	Hypertension (72.5%)	480 (86.5%)	911 (67.7%)		
				Hyperlipemia (78.3%)	504 (90.6%)	999 (74.5%)		
				Current smoker (21.4%)	104 (18.6%)	306 (22.5%)		
			Study design	Randomization	RCT			
				Blinding	Single-blind			
				Center	Multicenter, America			
				Endpoint of interest	TLR			
				Comparison	Ridaforolimus-eluting stents vs. zotarolimus-eluting stents			
				Follow-up	2 years			
				Artery	Coronary			
BIOSCIENCE [58] 2015	Patients with coronary artery disease and at least 1 lesion with a diameter stenosis > 50% (de novo or restenosis) in a native vessel or in bypass graft were eligible for inclusion	Not clear	Demographics	N	486	1633	Intraprocedural medications included unfractionated heparin (with a dose of 5000 IU or 70–100 IU/kg of body weight) or bivalirudin. Dual antiplatelet therapy was started before or at the time of PCI and consisted of acetylsalicylic acid (> 250 mg) in combination with clopidogrel (loading dose, 600 mg; maintenance dose, 75 mg QD), prasugrel (loading dose, 60 mg; maintenance dose, 10 mg QD), or ticagrelor (loading dose, 180 mg; maintenance dose, 90 mg BID) for the recommended duration of 12 months	Dual antiplatelet therapy consisted of acetylsalicylic acid (> 250 mg) in combination with clopidogrel (loading dose, 600 mg; maintenance dose, 75 mg QD), prasugrel (loading dose, 60 mg; maintenance dose, 10 mg QD), or ticagrelor (loading dose, 180 mg; maintenance dose, 90 mg BID) for the recommended duration of 12 months
				Age				
				Male sex (77.1%)	371 (76.3%)	1263 (77.3%)		
				Restenosis	29	44		
			Treatment	Oral medication	345 (71.0%)			
				Insulin	160 (32.9%)			
				Diet only	152 (31.3%)			
				No treatment	21 (4.3%)			
			Risk factors	Hypertension (67.7%)	409 (84.2%)	1025 (62.8%)		
				Hyperlipemia (67.4%)	361 (74.3%)	1067 (65.3%)		
				Current smoker (28.7%)	109 (22.4%)	500 (39.6%%)		
				Family history of CAD	127 (26.1%)	460 (36.4%)		
			Study design	Randomization	RCT			
				Blinding	Single-blind			
				Center	Multicenter, Europe			
				Endpoint of interest	TLR			
				Comparison	Biodegradable polymer sirolimus-eluting stents vs. durable polymer everolimus-eluting stents			
				Follow-up	1 year			
				Artery	Coronary			
CIBELES [59] 2010	Patients with total coronary occlusions with an estimated time since occlusion of > 2 weeks	Acute myocardial infarction at the area supplied by the target vessel within 2 weeks before the inclusion in the study; The lesion cannot be crossed with the guidewire and balloon angioplasty; The target lesion has been previously treated percutaneously; The lesion is not suitable for 2.25–3.5 mm coronary stent implantation; The patient is not willing to undergo angiographic follow-up; The patient has contraindications for prolonged double antiplatelet therapy; Pregnancy or absence of a negative pregnancy test result in women of childbearing age; Chronic renal failure; Plasma platelet count < 100.000 mm–3 or > 700.000 mm–3; The patient has any severe noncardiac disease that reduces his or her life expectancy to < 1 year; The patient is currently included in another randomized trial	Demographics	N	75	132	Unfractionated heparin will be administered IV during PCI	Not available
				Age	64.9 ± 9.2	63.8 ± 11.0		
				Male sex (84.1%)	60 (77.3%)	114 (86.4%)		
				Restenosis	3	14		
			Risk factors	Hypertension (68.1%)	53 (70.7%)	88 (66.7%)		
				Hyperlipemia (71.5%)	57 (76.0%)	91 (68.9%)		
				Current smoker (55.6%)	43 (57.3%)	72 (54.5%)		
			Study design	Randomization	RCT			
				Blinding	Single-blind			
				Center	Multicenter, Europe			
				Endpoint of interest	TLR			
				Comparison	Everolimus-eluting stents vs. sirolimus-eluting stents			
				Follow-up	9 months			
				Artery	Coronary			
ENDEAVOR IV [60] 2010	Consecutive adult patients with clinical evidence of ischemic coronary disease or a positive functional study were enrolled at 80 centers in the US	Key clinical exclusion criteria included recent acute MI, another planned PCI within the next 30 days or previous PCI in the target vessel within the previous 9 months, recent stroke or transient ischemic attack, left ventricular ejection fraction 30%, and contraindication to dual antiplatelet therapy (aspirin and a thienopyridine)	Demographics	N	477	1071	Patients were treated with 75 mg of aspirin within 24 h before the procedure	All patients received a loading dose of clopidogrel of at least 300 mg followed by 75 mg/day for at least 6 months and aspirin 75 mg indefinitely
				Age				
				Male sex (67.7%)	288 (60.38%)	760 (70.96%)		
				Restenosis	29	29		
			Treatment	Medications for diabetes	426 (89.31%)			
				No drugs	51 (10.69%)			
			Risk factors	Hypertension (81%)	432 (90.57%)	822 (76.75%)		
				Hyperlipemia (83.1%)	415 (87.00%)	871 (81.33%)		
				History of smoking (60.8%)	255 (53.46%)	686 (64.05%)		
			Study design	Randomization	RCT			
				Blinding	Single-blind			
				Center	Multicenter, America			
				Endpoint of interest	TLR			
				Comparison	Zotarolimus-Eluting vs. Paclitaxel-Eluting Stents			
				Follow-up	1 year			
				Artery	Coronary			
HORIZONS AMI [61] 2009	Consecutive patients 18 years of age or older who presented within 12 h after the onset of symptoms and who had ST-segment elevation of 1 mm or more in two or more contiguous leads, new left bundle-branch block, or true posterior myocardial infarction were considered for enrollment	The clinical exclusion criteria were contraindications to study medications, conditions that increase the risk of hemorrhage, and an inability to take clopidogrel for 6 months after the procedure	Demographics	N	533	2732	Aspirin (324 mg administered in chewable form or 500 mg administered intravenously) was given in the emergency room, after which 300 to 325 mg was given orally every day during the hospitalization A loading dose of clopidogrel (either 300 mg or 600 mg, at the discretion of the investigator) was administered before catheterization	A loading dose of aspirin 75 to 81 mg every day thereafter indefinitely, clopidogrel 75 mg orally every day for at least 6 months
				Age	64.1	59.4		
				Male sex (77.2%)	392 (73.5%)	2127 (77.9%)		
				Restenosis	30	122		
			Treatment	Oral medication	282 (52.9%)			
				Insulin	145 (27.2%)			
				Diet only	102 (19.1%)			
			Risk factors	Hypertension (41.9%)	379 (71.7%)	1329 (48.6%)		
				Hyperlipemia (42.9%)	318 (59.7%)	1082 (39.6%)		
				Current smoker (46.8%)	194 (36.5%)	1334 (49.1%)		
			Study design	Randomization	RCT			
				Blinding	Not blinded			
				Center	Multicenter, America			
				Endpoint of interest	TLR			
				Comparison	Paclitaxel-eluting stents vs. bare metal stents			
				Follow-up	1 year			
				Artery	Coronary			
ICE [62] 2017	Patients were eligible for inclusion if they had peripheral artery disease of Rutherford stage 1 to 4 due to a single significant common or external iliac artery lesion of 10 to 200 mm in length, not extending into the aorta or the common femoral artery. Sequential lesions at a distance of < 10 mm counted as a single lesion	Key exclusion criteria were dialysis dependent end-stage renal disease and treatment with oral anticoagulants other than antiplatelet agents	Demographics	N	164	496	Patients not on chronic antiplatelet therapy were premedicated with acetylsalicylic acid (100 mg/day) and clopidogrel (75 mg/day) for at least 10 days. Patients not on this regimen were administered an intravenous bolus of 500 mg acetylsalicylic acid and an oral loading dose of 600 mg clopidogrel before or immediately after the procedure. After sheath placement, a bolus of 5,000 U of heparin was administered	All patients had to be maintained on clopidogrel (75 mg/day) for at least 1 month and on acetylsalicylic acid (100 mg/day) indefinitely after the procedure
				Age	63.6			
				Male sex	497 (75.3%)			
				Restenosis	6	32		
			Treatment	Not available				
			Risk factors	Hypertension	485 (73.5%)			
				Hyperlipemia	416 (63%)			
				Current smoker	397 (60.2%)			
			Study design	Randomization	RCT			
				Blinding	Not blinded			
				Center	Multicenter, Europe			
				Endpoint of interest	QA-R			
				Comparison	Balloon-expandable stent vs. self-expanding stent			
				Follow-up	1 year			
				Artery	Femoropopliteal Arteries			
IMAP [63] 2011	The study included patients with stable angina or acute coronary syndrome (unstable angina or myocardial infarction) with de novo multivessel coronary artery lesions of ≥ 70% by quantitative coronary angiographic analysis who were suitable for stent implantation	Patients were excluded if there was failure to provide written informed consent, contraindication to any emergency myocardial revascularization surgery, patients with single-vessel disease, restenotic lesions, chronic total occluded lesions, significant left main disease, patients undergoing primary angioplasty, contraindications to the use of acetylsalicylic acid or clopidogrel and a left ventricular ejection fraction of < 30%. Patients with cardiogenic shock, malignancies or other comorbidities with life expectancy < 12 months or that may result in noncompliance with the protocol, or pregnancy were considered ineligible for the study	Demographics	N	68	119	Patients were treated with 200 mg of aspirin daily and 300 mg of clopidogrel on the day before the procedure. Isosorbide-5 mononitrate (20 mg) was administered intracoronarily before the analysis of quantitative coronary angiography (before and immediately after), and heparin at a dose of 100 IU/kg was administered intravenously before implantation	75 mg of clopidogrel was administered for 30 days and aspirin indefinitely
				Age	59.5			
				Male sex	124 (66.3%)			
				Restenosis	27	33		
			Risk factors	Hypertension	146 (78%)			
				Hyperlipemia	85 (45.5%)			
				Current smoker	71 (37.9%)			
				Family history of CAD	116 (62%)			
			Study design	Randomization	RCT			
				Blinding	Unclear			
				Center	Single center, America			
				Endpoint of interest	QA-R			
				Comparison	Cobalt–chromium stent vs. stainless steel stent implantation			
				Follow-up	6 months			
				Artery	Coronary			
ISAR-DESIRE 2 [64] 2020	Patients > 18 years of age with ischemic symptoms or evidence of myocardial ischemia (inducible or spontaneous) in the presence of a restenosis ≧50% located in the native vessel segment treated with SES were considered eligible	Patients with a target lesion located in the left main stem, acute myocardial infarction within the preceding 48 h, cardiogenic shock, malignancies, or other comorbid conditions with life expectancy < 12 months or that may result in protocol noncompliance, known allergy to the study medications (sirolimus, paclitaxel), or pregnancy (present, suspected, or planned) were considered ineligible for the study	Demographics	N	154	288	An oral loading dose of 600 mg clopidogrel was administered to all patients before the intervention, regardless of whether the patient was receiving clopidogrel before admission. During the procedure, patients were given intravenous aspirin, heparin, or bivalirudin	After the intervention, all patients, irrespective of treatment allocation, were prescribed 200 mg/day aspirin indefinitely, clopidogrel 150 mg for the first 3 days (or until discharge) followed by 75 mg/day for at least 6 months
				Age				
				Male sex (6.79%)	10 (6.49%)	20 (6.94%)		
				Restenosis	36	52		
			Risk factors	Hypertension (73.8%)	120 (77.92%)	206 (71.5%)		
				Hyperlipemia (77.1%)	129 (83.8%)	212 (73.6%)		
				Current smoker (12.2%)	12 (7.8%)	42 (14.6%)		
			Study design	Randomization	RCT			
				Blinding	Double-blind			
				Center	Multicenter, Europe			
				Endpoint of interest	QA-R			
				Comparison	Sirolimus-eluting stent vs. paclitaxel-eluting stent			
				Follow-up	8 mouths			
				Artery	Coronary			
ISAR-TEST 4 [65] 2021	Patients older than age 18 years with ischemic symptoms or evidence of myocardial ischemia (inducible or spontaneous) in the presence of ≥ 50% de novo stenosis located in native coronary vessels were enrolled	Patients with a target lesion located in the left main stem or in cardiogenic shock were considered ineligible for the study	Demographics	N	560	1391	An oral loading dose of 600 mg clopidogrel was administered to all patients at least 2 h prior to the intervention, regardless of whether the patient was taking clopidogrel prior to admission. During the procedure, patients were given intravenous aspirin, heparin, or bivalirudin	After the intervention, all patients, irrespective of treatment allocation, were prescribed 200 mg/day aspirin indefinitely, clopidogrel 150 mg for the first 3 days (or until discharge) followed by 75 mg/day for at least 6 months
				Age	67.1	65.5		
				Male sex (76.1%)	413 (73.8%)	1072 (77.1%)		
				Restenosis	109	219		
			Treatment	Oral medication	286 (51.1%)			
				Unknown	274 (48.9%)			
			Risk factors	Hypertension (68.6%)	431 (77.0%)	908 (65.3%)		
				Hyperlipemia (66.2%)	374 (66.8%)	917 (65.9%)		
				Current smoker (15.5%)	74 (13.2%)	229 (16.5%)		
			Study design	Randomization	RCT			
				Blinding	Single-blind			
				Center	Multicenter, Europe			
				Endpoint of interest	TLR			
				Comparison	Biodegradable polymer drug-eluting stent vs. permanent polymer drug-eluting stent			
				Follow-up	10 years			
				Artery	Coronary			
ISAR-TEST 5 [66] 2021	It enrolled patients older than 18 years of age with ischemic symptoms or evidence of myocardial ischemia (inducible or spontaneous) in the presence of written, informed consent by the patient or her or his legally authorized representative for participation in the study was obtained	Patients with a target lesion located in the left main stem, cardiogenic shock, malignancies or other comorbid conditions with life expectancy less than 12 months or that may result in protocol noncompliance, known allergy to the study medications (probucol, sirolimus, zotarolimus) or pregnancy (present, suspected or planned) were considered ineligible for the study	Demographics	N	870	2132	An oral loading dose of 600-mg clopidogrel was administered to all patients at least 2 h before the intervention, regardless of whether the patient was taking clopidogrel before being admitted. During the procedure, patients were given intravenous aspirin, heparin, or bivalirudin	After the intervention, all patients, irrespective of treatment allocation, were prescribed 200 mg/d aspirin indefinitely, clopidogrel 150 mg for the first 3 days (or until discharge) followed by 75 mg/d for at least 6 months
				Age				
				Male sex (76.45%)	641	1645		
				Restenosis	192	354		
			Treatment	Oral medication	438 (50.3%)			
				Insulin	288 (33.1%)			
				Unknown	144 (16.6%)			
			Risk factors	Hypertension (66.7%)	637 (73.2%)	1365 (64.0%)		
				Hyperlipemia (63.5%)	577 (66.3%)	1330 (62.4%)		
				Current smoker (17.4%)	157 (18.0)	366 (17.2%)		
			Study design	Randomization	RCT			
				Blinding	Double-blind			
				Center	Multicenter, Europe			
				Endpoint of interest	TLR			
				Comparison	Polymer-free sirolimus- and probucol-eluting stents vs. permanent polymer zotarolimus-eluting stents			
				Follow-up	10 years			
				Artery	Coronary			
SIRIUS [67] 2004	Selection criteria included patients who were willing and able to comply with the requirements of the protocol who had a history of angina and signs of myocardial ischemia clinically correlated with a de novo target lesion of 50% diameter stenosis, 15 to 30 mm in length, in a native coronary artery of 2.5 to 3.5 mm diameter based on angiographic visual estimates	Major exclusion criteria included (1) myocardial infarction within the previous 24 h, (2) left ventricular ejection fraction 25%, (3) a target lesion located in an ostial or bifurcation location or one that had a thrombotic or severely calcified appearance, or (4) significant (50% diameter) stenosis within the target coronary artery proximal or distal to the target lesion	Demographics	N	275	783	All patients received oral aspirin (325 mg daily) and clopidogrel (loading dose of 300 to 375 mg, commencing 24 h before the index procedure. Intraprocedural intravenous heparin was given to maintain an activated clotting time of ≥ 250 s	Aspirin (325 mg daily) and clopidogrel (loading dose of 75 mg for 3 months)
				Age	62.3			
				Male sex	751 (71%)			
				Restenosis	42	65		
			Risk factors	Hypertension	719 (68%)			
				Hyperlipemia	783 (74%)			
				Current smoker	211 (20%)			
			Study design	Randomization	RCT			
				Blinding	Double-blind			
				Center	Multicenter, Europe			
				Endpoint of interest	TLR			
				Comparison	Sirolimus-eluting stents vs. bare metal stents			
				Follow-up	9 months			
				Artery	Coronary			
SIRTAX [68] 2012	Eligible patients had a history of stable angina or acute coronary syndrome and presented with at least one lesion with a diameter stenosis ≥ 50% in a vessel with a reference vessel diameter (RVD) between 2.25 and 4.00 mm suitable for stent implantation	Unclear	Demographics	N	201	811	Before or at the time of the procedure, patients received at least 100 mg of aspirin, a 300 mg loading dose of clopidogrel, and unfractionated heparin (70–100 U/kg of body weight)	After the procedure, all patients were advised to maintain aspirin lifelong, and clopidogrel therapy was prescribed for 12 months irrespective of stent type
				Age	65.9	61.4		
				Male sex (76.98%)	140 (70.7%)	639 (78.8%)		
				Restenosis	24	81		
			Risk factors	Hypertension (51.5%)	162 (80.6%)	460 (50.7%)		
				Hyperlipemia (59%)	123 (61.2%)	474 (58.5%)		
				Current smoker (36.1%)	41 (20.4%)	324 (40%)		
			Study design	Randomization	RCT			
				Blinding	Single-blind			
				Center	Multicenter, Europe			
				Endpoint of interest	TLR			
				Comparison	Sirolimus-eluting stents vs. paclitaxel-eluting stents			
				Follow-up	5 years			
				Artery	Coronary			
SORT OUT III [69] 2011	The trial included patients ≥ 18 years old with stable chronic coronary artery disease or acute coronary syndromes. Patients were eligible if they had ≥ 1 target lesion defined as a lesion to be treated with a drug-eluting stent	Exclusion criteria were inability to provide informed consent, life expectancy of < 1 year, allergy to acetylsalicylic acid, clopidogrel, ticlopidine, sirolimus, or zotarolimus, or participation in another randomized trial	Demographics	N	337	1995	Patients were pretreated with acetylsalicylic acid ≥ 75 mg, clopidogrel 300 to 600 mg loading dose, and unfractionated heparin (5,000 IU or 70 to 100 IU/kg)	Dual antiplatelet regimens included recommendations of lifelong acetylsalicylic acid (75 mg/day) and clopidogrel (75 mg/day) for 1 year
				Age	66	64		
				Male sex (73.5%)	241 (71.5%)	1473(73.8%)		
				Restenosis	23	68		
			Risk factors	Hypertension (50.3%)	236 (70.2%)	938 (47.0%)		
				Hyperlipemia (66%)	263 (78.0%)	1275 (63.9%) (63.91%)		
				Current smoker (29.5%)	89 (26.41%)	599 (30.0%)		
			Study design	Randomization	RCT			
				Blinding	Not blinded			
				Center	Multicenter, Europe			
				Endpoint of interest	TLR			
				Comparison	Zotarolimus-eluting stents vs. sirolimus-eluting stents			
				Follow-up	1 year			
				Artery	Coronary			
SORT OUT IV [70] 2012	Patients were eligible if they were ≥ 18 years old, had chronic stable coronary artery disease or acute coronary syndromes, and ≥ 1 coronary lesion with ≥ 50% diameter stenosis	Exclusion criteria were life expectancy of < 1 year; allergy to aspirin, clopidogrel, sirolimus, or everolimus; participation in another randomized trial; or inability to provide written informed consent	Demographics	N	390	2384	Before or at the time of the procedure, patients received aspiring ≥ 75 mg, clopidogrel 600-mg loading dose, and an unfractionated heparin dose (5,000 IU or 70 to 100 IU/kg)	Recommended postprocedure dual antiplatelet regimens were aspirin 75 mg/day lifelong and clopidogrel 75 mg/day for 1 year
				Age	63.6	64.3		
				Male sex (75.56%)	290 (74.4%)	1806 (75.6%)		
				Restenosis	21	50		
			Risk factors	Hypertension (48.2%)	273 (70.0%)	1065 (44.7%)		
				Hyperlipemia (62.2%)	305 (78.2%)	1420 (59.6%)		
				Current smoker (25.1%)	89 (22.8%)	608 (25.5%)		
			Study design	Randomization	RCT			
				Blinding	Single-blind			
				Center	Multicenter, Europe			
				Endpoint of interest	TLR			
				Comparison	Everolimus-eluting stent vs. sirolimus-eluting stent			
				Follow-up	18 months			
				Artery	Coronary			
SORT OUT VIII [71] 2019	Patients were eligible if they had chronic stable coronary artery disease or acute coronary syndromes, including myocardial infarction (MI) with or without ST-segment elevation, and at least 1 coronary or vein graft lesion with more than 50% diameter stenosis in a vessel with a reference diameter of minimum 2.25 mm	Exclusion criteria were life expectancy of < 1 year; allergy to aspirin, P2Y12 platelet inhibitors, everolimus, or biolimus; clinical indication of inability to tolerate dual-antiplatelet treatment for 12 months; and inability to provide written informed consent	Demographics	N	512	2252	Before implantation, patients received at least 75 mg of aspirin and a loading dose of a P2Y12 platelet inhibitor (600 mg clopidogrel, 180 mg ticagrelor, or 60 mg prasugrel) orally and unfractionated heparin intravenously (5,000 to 10,000 IU or 70 to 100 IU/kg)	Recommended postprocedural dual-antiplatelet regimens were aspirin 75 mg/day lifelong and a P2Y12 platelet inhibitor for 12 months
				Age	66.9	66.2		
				Male sex (76.56%)	374 (73.0%)	1742 (77.4%)		
				Restenosis	15	52		
			Treatment	Oral medication	264 (51.6%)			
				Insulin	166 (32.4%)			
				Diet only	34 (6.6%)			
			Risk factors	Hypertension (56.9%)	395 (77.1%)	1177 (52.3%)		
				Hyperlipemia (53.3%)	384 (75.0%)	1088 (48.3%)		
				Current smoker (29.1%)	122 (23.8%)	681 (30.2%)		
			Study design	Randomization	RCT			
				Blinding	Unknown			
				Center	Multicenter, Europe			
				Endpoint of interest	TLR			
				Comparison	Everolimus-eluting stent (EES) with the biolimus-eluting stainless-steel stent (BES)			
				Follow-up	1 year			
				Artery	Coronary			
TAXUS IV [72] 2009	Patients who were at least 18 years of age, had stable or unstable angina or provokable ischemia, and were undergoing percutaneous coronary intervention for a single, previously untreated lesion in a native coronary artery were considered for enrollment	Clinical exclusion criteria included previous or planned use of intravascular brachytherapy in the target vessel or of any drug-eluting stent; myocardial infarction within 72 h before enrollment; a left ventricular ejection fraction of less than 25 percent; hemorrhagic diatheses; contraindications or allergy to aspirin, thienopyridines, paclitaxel, or stainless steel; a history of anaphylaxis in response to iodinated contrast medium; use of paclitaxel within 12 months before study entry or current use of colchicine; a serum creatinine level of more than 2.0 mg per deciliter, a leukocyte count of less than 3500 per cubic millimeter, or a platelet count of less than 100,000 per cubic millimeter; a recent positive pregnancy test, breast-feeding, or the possibility of a future pregnancy; coexisting conditions that limited life expectancy to less than 24 months or that could affect a patient’s compliance with the protocol; and current participation in other investigational trials	Demographics	N	318	996	Patients were pretreated with aspirin 325 mg and clopidogrel 300 mg before the procedure, and unfractionated heparin was administered per standard practice	Treated with aspirin 325 mg daily indefinitely and clopidogrel 75 mg daily for at least 6 months
				Age	62.2	62.6		
				Male sex (72.1%)	202 (63.5%)	745 (74.8%)		
				Restenosis	64	126		
			Treatment	Insulin	105 (33.0%)			
				Unknown	213			
			Risk factors	Hypertension (69.9%)	256 (81.1%)	663 (66.6%)		
				Hyperlipemia (66.3%)	227 (71.4%)	644 (64.7%)		
			Study design	Randomization	RCT			
				Blinding	Double-blind			
				Center	Single center, America			
				Endpoint of interest	TLR			
				Comparison	Bare-metal stent vs. paclitaxel-eluting stent			
				Follow-up	6 months			
				Artery	Coronary			
TOSCA [73] 2005	Patients 18 to 80 years old undergoing clinically indicated coronary interventions were eligible provided that 1 target segment met the study definition of occlusion: a high-grade native coronary stenosis accompanied by TIMI grade 0 or 1 antegrade flow	Exclusion criteria were (1),72 h since onset of ST-segment elevation, (2) extensive lesion-related thrombus (TIMI thrombus grade 3 or 4, Appendix 2), (3) occlusions previously revascularized by patent bypass grafts, (4) uncontrolled heart failure or shock, (5) patient unwilling or unsuitable for protocol-required 6-month angiography, (6) patient of child-bearing potential, and (7) inability to cross occlusion with guidewire	Demographics	N	68	342	All patients received aspirin 325 mg/d before the procedure	Unknown
				Age				
				Male sex (89.3%)	48 (70.6%%)	318 (93.0%)		
				Restenosis	45	213		
			Risk factors	Hypertension (38.8%)	35 (51.5%)	124(36.3%)		
				Current smoker (18.8%)	8 (11.8%)	69 (20.2%)		
			Study design	Randomization	RCT			
				Blinding	Not blinded			
				Center	Multicenter, America			
				Endpoint of interest	QA-R			
				Comparison	Stenting vs. balloon angioplasty alone			
				Follow-up	6 months			
				Artery	Coronary			
ZEST [74] 2012	We sought to enroll consecutive patients aged 18 years or older with either stable angina or acute coronary syndromes who had at least 1 coronary lesion (defined as stenosis of more than 50%) suitable for stent implantation	Exclusion criteria were ST-segment elevation MI necessitating primary PCI; severely compromised ventricular dysfunction (ejection fraction < 25%) or cardiogenic shock; allergy to antiplatelet drugs, heparin, stainless steel, contrast agents, zotarolimus, sirolimus, or paclitaxel; left main coronary artery disease (defined as stenosis of more than 50%); in-stent restenosis of drug-eluting stents; terminal illness; and participation in another coronary-device study	Demographics	N	760	1885	All patients received at least 100 mg of aspirin and a 300- to 600-mg loading dose of clopidogrel before or during the procedure. Heparin was administered throughout the procedure to maintain an activated clotting time of 250 s or longer	Patients received 100 mg/day of aspirin continuously and 75 mg/day clopidogrel for at least 12 months after the procedure
				Age				
				Male sex (66.5%)	462 (60.8%)	1297 (68.8%)		
				Restenosis	37	104		
			Treatment	Insulin	101 (13.3%)			
				Unknown	659			
			Risk factors	Hypertension (60.8%)	549 (72.2%)	1060 (56.2%)		
				Hyperlipemia (51.5%)	369 (48.6%)	994 (52.7%)		
				Current smoker (27.8%)	188 (24.7%)	547 (29%)		
				Family history of CAD	34 (4.5%)	110 (%)		
			Study design	Randomization	RCT			
				Blinding	Single-blind			
				Center	Single center, Asia			
				Endpoint of interest	TLR			
				Comparison	Zotarolimus-eluting stents vs. sirolimus-eluting stents (SES), and paclitaxel-eluting stents (PES)			
				Follow-up	2 years			
				Artery	Coronary			

*CAD* coronary artery disease. *FU-T* follow-up time. *DAPT* dual antiplatelet therapy. *MACE(s)* major adverse cardiac event(s). *QA-R* quantitative angiography-confirmed restenosis. *RCT* randomized controlled trial

### Description of the prospective randomized pooled trials and quality assessment of the included studies

**Fig. 2:**
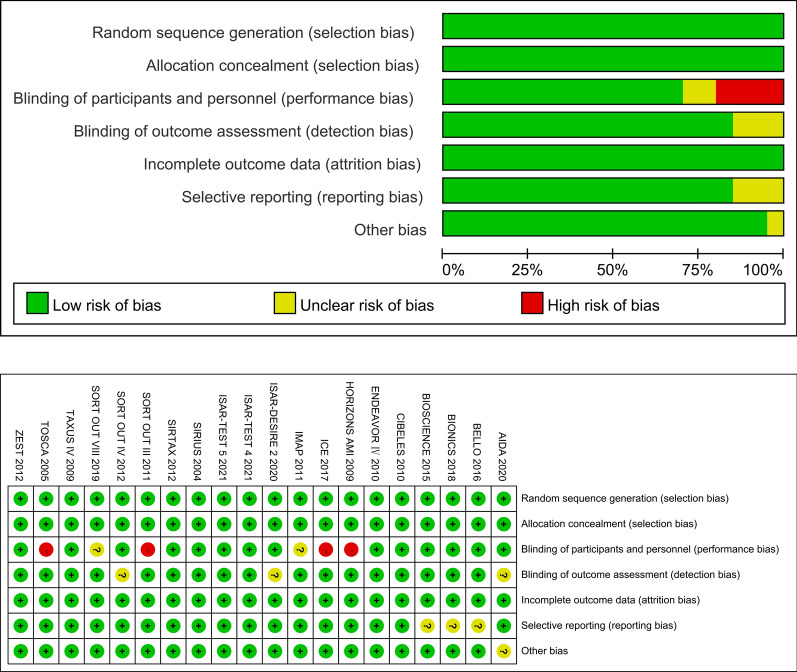
Risk of bias evaluated by the RoB 2 tool. All trials met the criteria and had a low risk of bias according to the Cochrane tool for assessing the risk of bias in RCTs

As the aim of this study was to analyze the PTA- or stenting-related outcomes among patients who suffered from cardiovascular stenotic or occlusive disease with or without DM, the efficacy endpoint was the rate of restenosis (> 50% stenosis at angiography or TLR during the follow-up period). As a result, 31,066 patients were enrolled in this study. Table 1 summarizes the characteristics of the 20 included RCTs. All trials met the inclusion criteria and had a low risk of bias according to the Cochrane tool [16] for assessing risk of bias in RCTs (Fig. 2). Four studies (TOSCA, SORT OUT III, ICE, HORIZONS AMI) were designed to be open-labeled, and the BASKET-SMALL 2 study did not describe its randomization method (Table 2).

**Table 2:** Blood glucose control and evaluation form

Study	Hypoglycemic treatment	Glycemic control levels	Follow-up times	Operational style
AIDA	Oral medication 196 (60.49%) Insulin 110 (33.95%) None 18 (5.56%) Unknown 4 (1.23%)	Under control	3 years	stent
BELLO	Insulin 25 (33.78%)	Unknown	3 years	PTA vs. Stent
BIONICS	Unknown	Unknown	2 years	Stent
BIOSCIENCE	Oral medication 345 (71.0%) Insulin 160 (32.9%) Diet only 152 (31.3%) None 21 (4.3%)	Under control	1 year	Stent
CIBELES	Unknown	Unknown	9 months	Stent
ENDEAVOR IV	Oral medication 426 (89.31%) None 51 (10.69%)	Under control	1 year	Stent
HORIZONS AMI	Oral medication 282 (52.9%) Insulin 145 (27.2%) Diet only 102 (19.1%)	Under control	1 year	Stent
ICE	Unknown	Unknown	1 year	Stent
IMAP	Unknown	Unknown	6 months	Stent
ISAR-DESIRE 2	Unknown	Unknown	8 months	Stent
ISAR-TEST 4	Oral medication 286 (51.1%) Unknown 274 (48.9%)	Unknown	10 years	Stent
ISAR-TEST 5	Oral medication 438 (50.3%) Insulin 288 (33.1%) Unknown 144 (16.6%)	Under control	10 years	Stent
SIRIUS	Unknown	Unknown	9 months	Stent
SIRTAX	Unknown	Unknown	5 years	Stent
SORT OUT III	Unknown	Unknown	1 year	Stent
SORT OUT IV	Unknown	Unknown	18 months	Stent
SORT OUT VIII	Oral medication 264 (51.6%) Insulin 166 (32.4%) Diet only 34 (6.6%)	Under control	1 year	Stent
TAXUS IV	Insulin 105 (33%) Unknown 213	Unknown	6 months	Stent
TOSCA	Unknown	Unknown	6 months	PTA vs. Stent
ZEST	Insulin 101 (13.3%) Unknown 659	Unknown		Stent

### Meta-analysis results

**Fig. 3:**
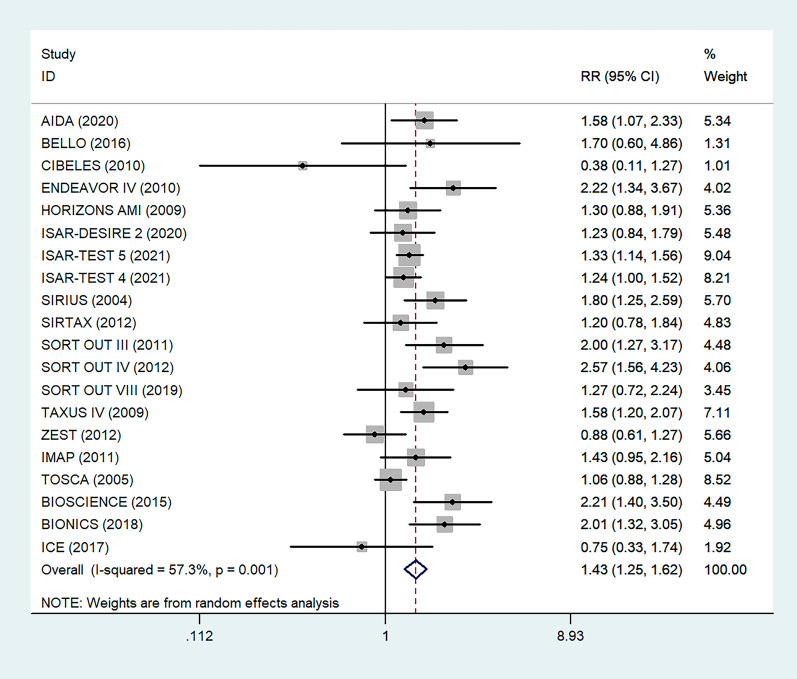
Forest plot of the association between DM and restenosis. The vertical dashed lines indicate the pooled summary estimate (95% CI) for all studies in Fig. 3 (95% CI, 1.25–1.62; I^2^= 57.53%, P = 0.001). The area of each square is proportional to the inverse variance of the estimate. The horizontal lines indicate the 95% confidence intervals of the estimate

There were 20 RCTs that included a total of 31,066 patients that reported data on DM and restenosis. Since there was high heterogeneity (*I*^*2*^ = *57.3%, P* = *0.001)*, a meta-analysis was conducted through a random effects model. Overall, the pooled results showed that DM was significantly associated with a higher risk of a major endpoint (RR = 1.43, 95% CI: 1.25–1.62; Fig. 3). The heterogeneity test showed significant differences among individual studies (P < 0.01, I^2^ = 57.3%). The sensitivity analysis showed that heterogeneity mainly came from BIONICS (2018), but the outcome did not change with removal of this study (RR 1.40,95% CI: 1.23–1.59) (Fig. 9).

### Subgroup analysis

**Fig. 4:**
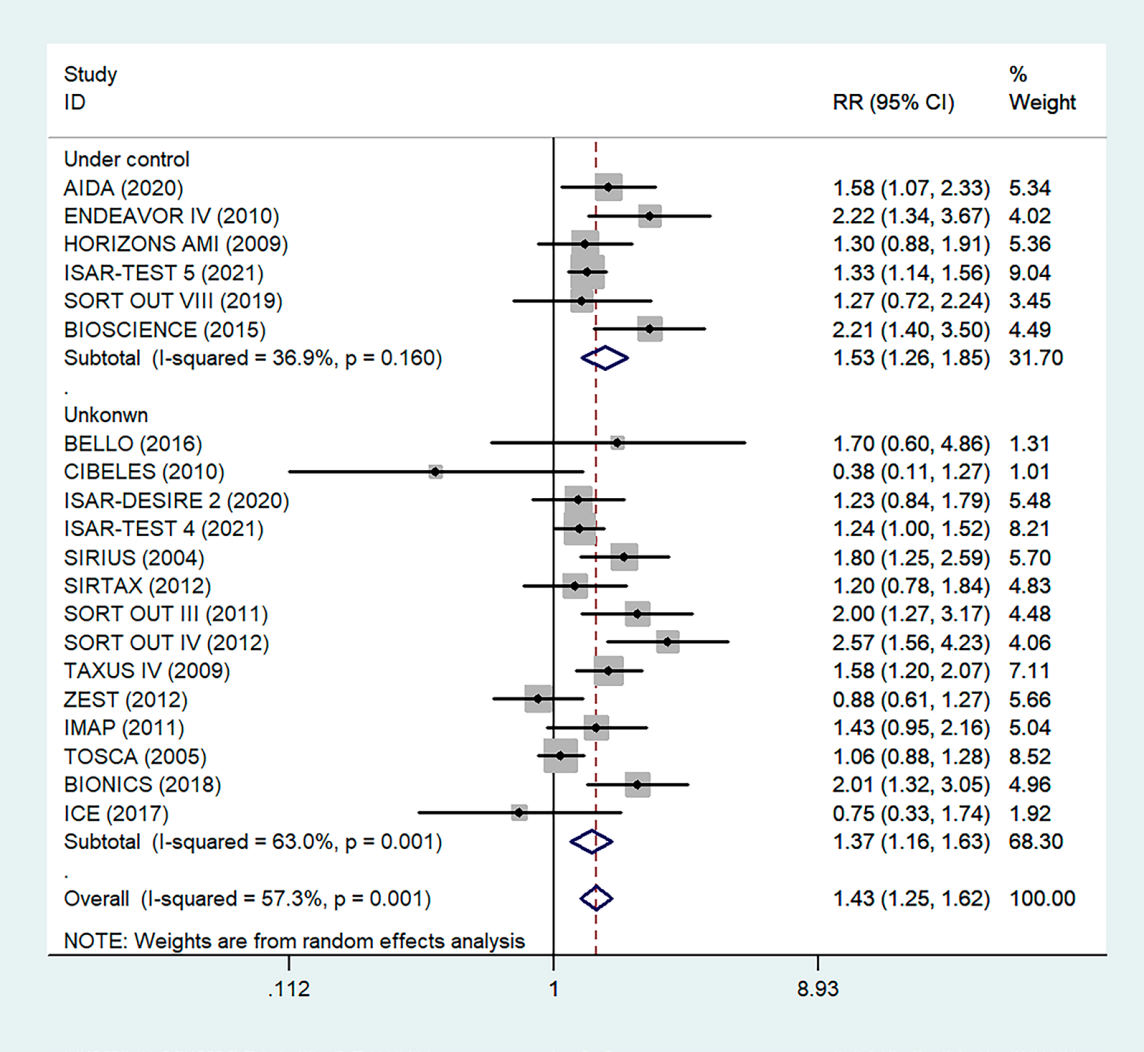
Subgroup analysis of the association between restenosis and glycemic control levels. The vertical dashed lines indicate the pooled summary estimate (95% CI) for all studies in Fig. 4 (‘Under Control’ subgroup, RR = 1.53, 95% CI, 1.25–1.62; I^2^ = 36.9%, P < 0.05, ‘Unknown’ subgroup, RR = 1.37, 95% CI, 1.16–1.63; I^2^ = 63%, P = 0.001.). The area of each square is proportional to the inverse variance of the estimate. The horizontal lines indicate the 95% confidence intervals of the estimate

To determine the relationship between glycemic control levels and the incidence of postoperative restenosis, we performed a subgroup analysis of the included studies. Since detailed blood glucose levels of patients could not be obtained, we divided the included studies into the "under control" group and the "unknown" group according to whether the proportion of overall medicine glycemic control (oral hypoglycemic agents or insulin) of diabetic patients in each study was more than 80% (Fig. 4). As a result, there was no significant difference in restenosis rates between the under-control group (RR = 1.53, 95% CI 1.26–1.84, P < 0.05) and the unknown group (RR = 1.37, 95% CI 1.16–1.63, P = 0.001).

**Fig. 5:**
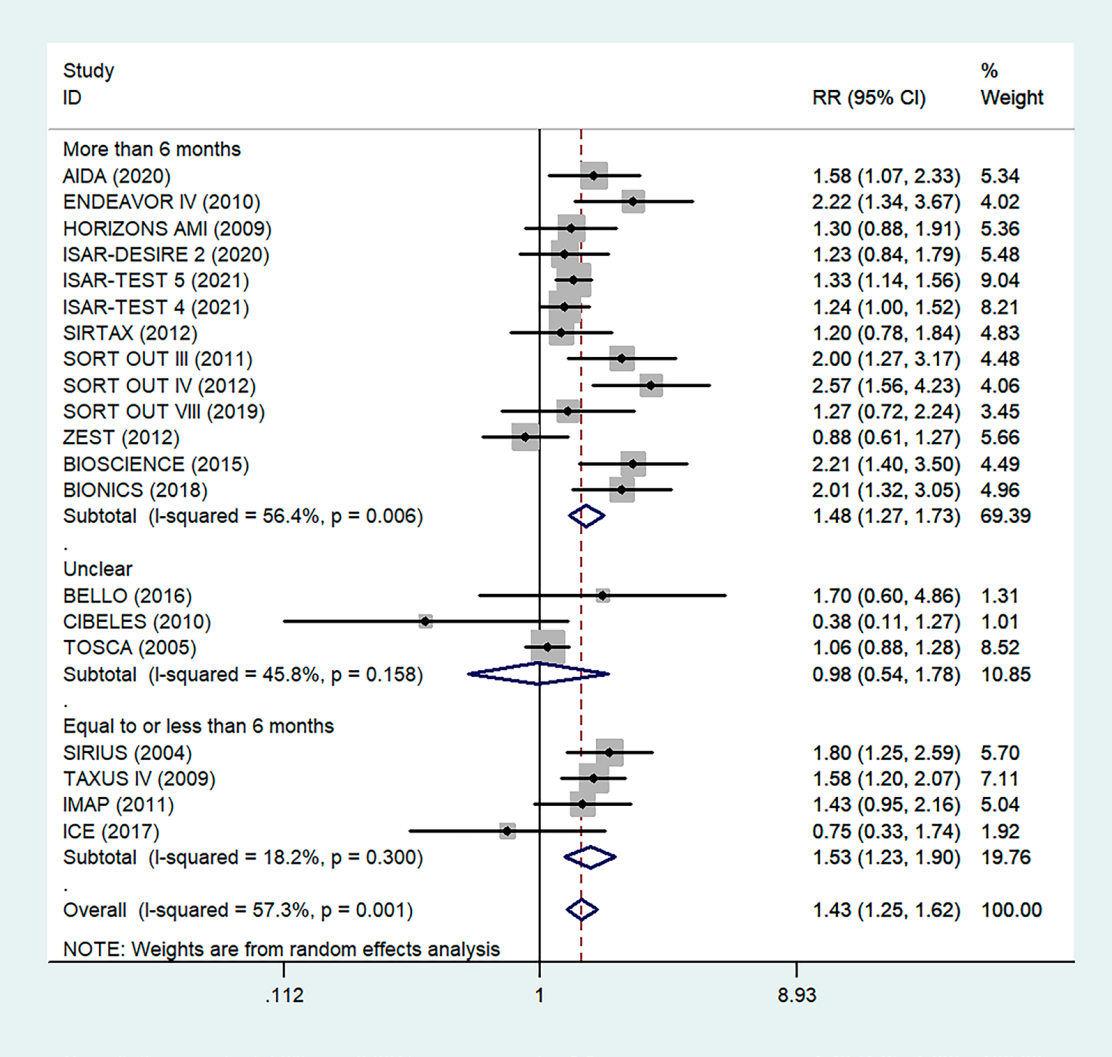
Subgroup analysis of the association between restenosis and the duration of DAPT. The vertical dashed lines indicate the pooled summary estimate (95% CI) for all studies in Fig. 5 (‘More than 6 months’ subgroup, RR = 1.48, 95% CI, 1.27–1.73; I^2^ = 56.4%, P = 0.006, ‘Equal to or less than 6 months’ subgroup, RR = 1.53, 95% CI, 1.23–1.90; I^2^ = 18.2%, P < 0.05.). The area of each square is proportional to the inverse variance of the estimate. The horizontal lines indicate the 95% confidence intervals of the estimate

**Fig. 6:**
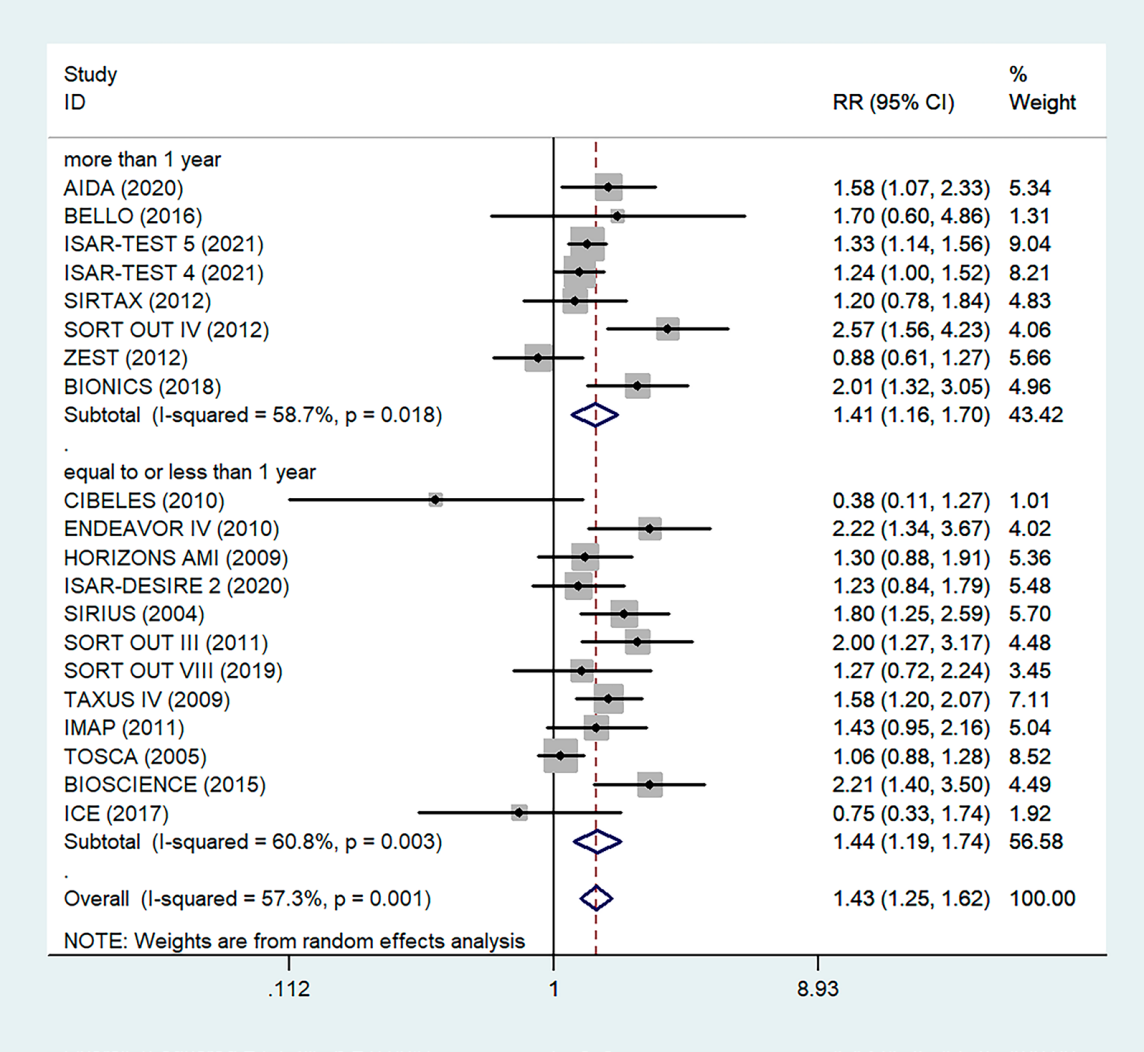
Subgroup analysis of the association between restenosis and different follow-up times. The vertical dashed lines indicate the pooled summary estimate (95% CI) for all studies in Fig. 6 (‘More than 1 year’ subgroup, RR = 1.41, 95% CI, 1.16–1.70; I^2^ = 58.7%, P = 0.018, ‘Equal to or less than 1 year’ subgroup, RR = 1.44, 95% CI, 1.19–1.74; I^2^ = 60.8%, P = 0.003.). The area of each square is proportional to the inverse variance of the estimate. The horizontal lines indicate the 95% confidence intervals of the estimate

**Fig. 7:**
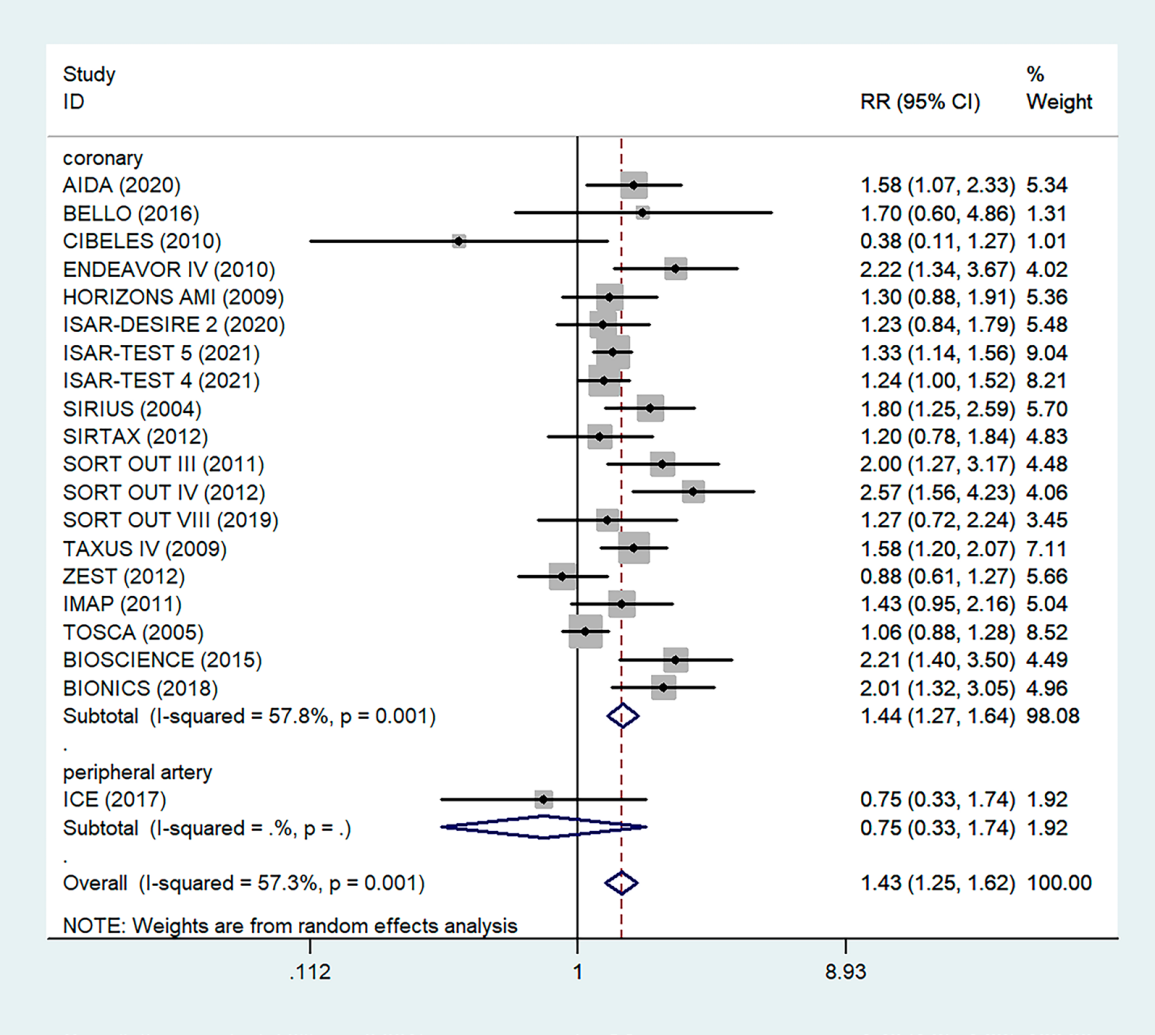
Subgroup analysis of the association between restenosis and types of lesion vessels. The vertical dashed lines indicate the pooled summary estimate (95% CI) for all studies in Fig. 7 (‘Coronary’ subgroup, RR = 1.44, 95% CI, 1.27–1.64; I^2^ = 57.8%, P = 0.001, ‘Peripheral’ subgroup, RR = 0.75, 95% CI, 0.33–1.74.). The area of each square is proportional to the inverse variance of the estimate. The horizontal lines indicate the 95% confidence intervals of the estimate

**Fig. 8:**
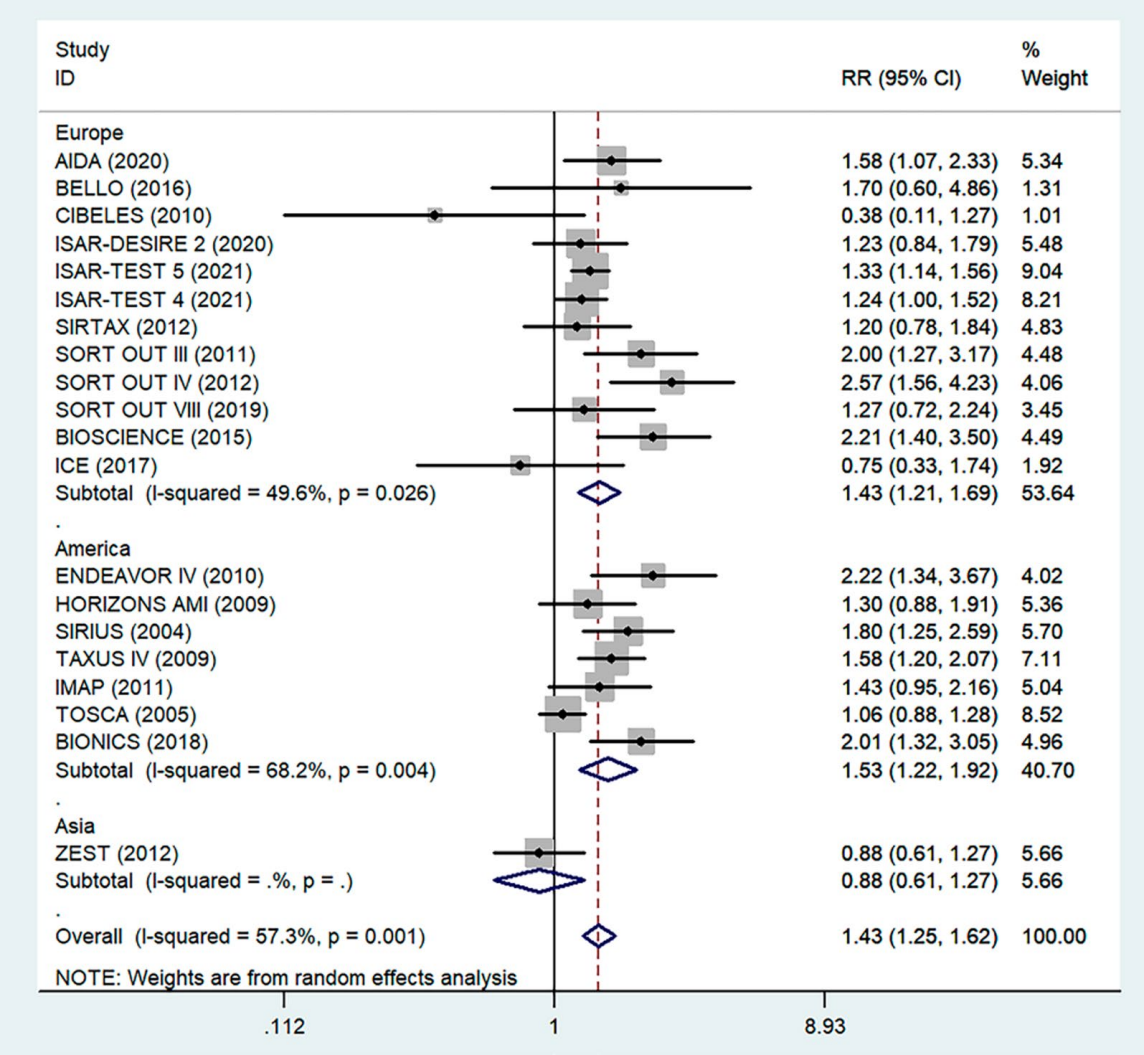
Subgroup analysis of the association between restenosis and different continents. The vertical dashed lines indicate the pooled summary estimate (95% CI) for all studies in Fig. 8 (‘Europe’ subgroup, RR = 1.43, 95% CI, 1.21–1.69; I^2^ = 49.6%, P < 0.05. ‘America’ subgroup, RR = 1.53, 95% CI, 1.22–1.92; I^2^ = 68.2%, P = 0.004.). The area of each square is proportional to the inverse variance of the estimate. The horizontal lines indicate the 95% confidence intervals of the estimate

**Fig. 9:**
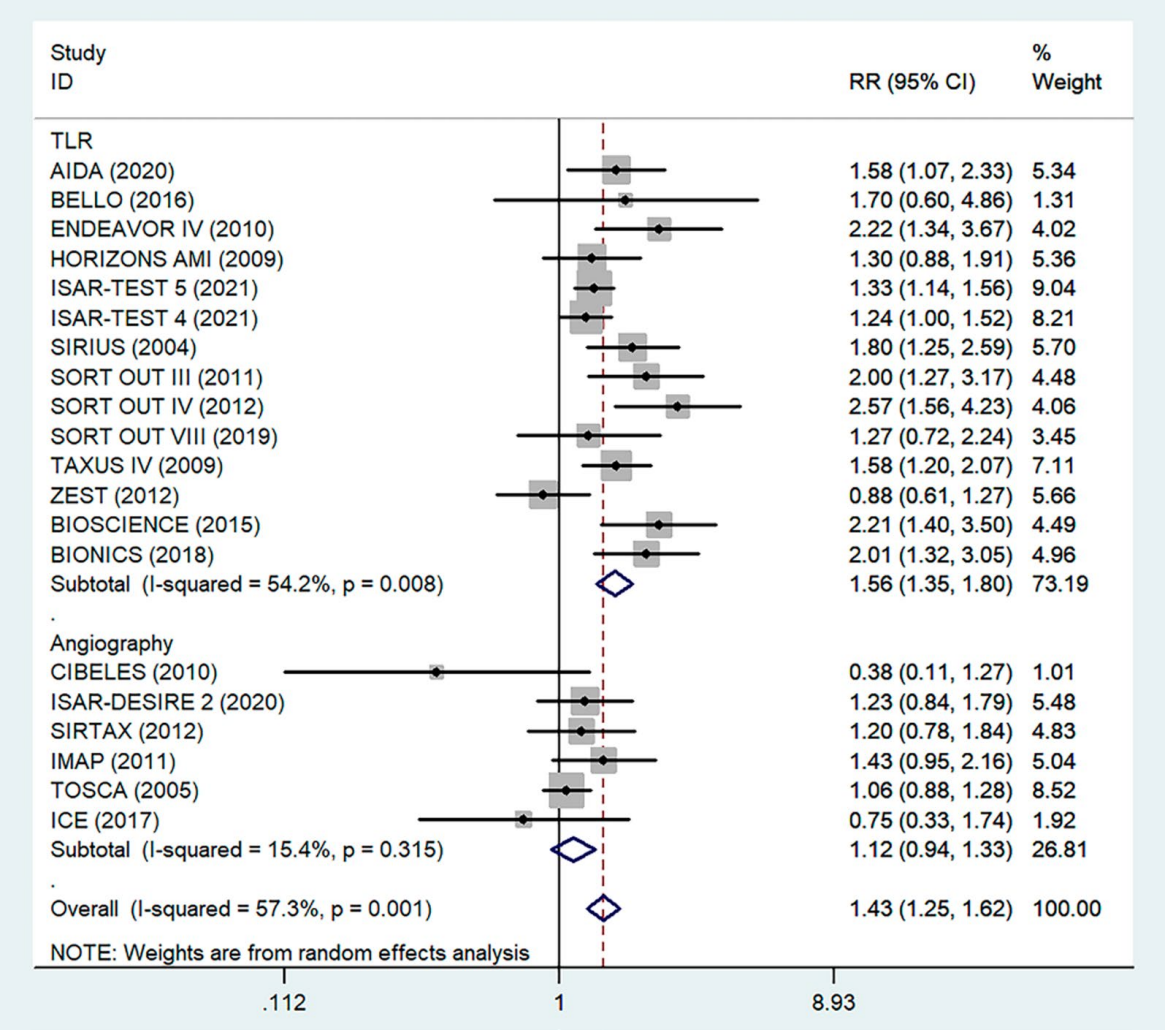
Subgroup analysis of the association between restenosis and diagnostic methods. The vertical dashed lines indicate the pooled summary estimate (95% CI) for all studies in Fig. 9 (‘TLR’ subgroup, RR = 1.56, 95% CI, 1.35–1.80; I^2^ = 49.6%, P = 0.008, ‘Angiography’ subgroup, RR = 1.12, 95% CI, 0.94–1.33; I^2^ = 15.4%, P = 0.315.). The area of each square is proportional to the inverse variance of the estimate. The horizontal lines indicate the 95% confidence intervals of the estimate

Antiplatelet therapy and anticoagulant therapy after vascular intervention are closely related to long-term efficacy [17]. Studies have demonstrated that an adequate treatment course of antiplatelet therapy after coronary stenting or angioplasty of lower extremity peripheral arteries is beneficial to improve long-term patency rates [18, 19]. We also analyzed the relationship between the postoperative dual antiplatelet therapy (DAPT) duration and restenosis in the studies. The results showed that regardless of the duration of DAPT, either less than (RR = 1.53, 95% CI 1.23–1.90, P < 0.05) or more than 6 months (RR = 1.48, 95% CI 1.27–1.73, P = 0.006) had no effect on restenosis outcome (Fig. 5). In addition, subgroup analysis was performed based on a 1-year follow-up. The results showed that there was no significant difference in the incidence of restenosis based on follow-up duration in the more than 1 year group (RR = 1.41, 95% CI 1.16–1.70, P = 0.018) and the less than or equal to 1 year group (RR = 1.44, 95% CI 1.19–1.74, P = 0.003) (Fig. 6). Furthermore, in these 20 studies, the major target vessel was the coronary artery, except for the ICE study, which evaluated peripheral arteries. Then, we performed a subgroup analysis by different target lesions. As shown in the forest plot, the restenosis rate after endovascular therapy was related to the primary site of the vascular lesion (coronary artery (RR = 1.44, 95% CI 1.27–1.64) or lower limb artery (RR = 0.75, 95% CI 0.33–1.74)) (Fig. 7). Moreover, subgroup analysis was also performed to account for continental differences in the included populations. Since there was only one study conducted among Asians, our subgroup included European and American participants (without distinguishing between South and North America), and it showed no significant differences in the endpoints between Americans (RR = 1.53, 95% CI 1.22–1.92, P = 0.004) and Europeans (RR = 1.43, 95% CI 1.21–1.69, P < 0.05) (Fig. 8). According to the diagnosis of restenosis, including angiography and TLR, we conducted a subgroup analysis of the two modalities, and the results showed that patients with diabetes had a higher risk of TLR (RR = 1.53, 95% CI 1.34–1.76, P = 0.010) (Fig. 9).

### Quantified covariable analysis-meta-regression analyses

With the aim of performing a comprehensive literature review on restenosis after interventional endovascular treatments of PTA or stenting in patients with diabetes (1) across different “interventions” (we performed a regression analysis according to different interventional modalities, i.e., aspirin, PTA or stent placement) (2) among patients with different health conditions (the proportion of patients with smoking exposure, hypertension, or hyperlipemia, was distinguished and regression analysis was performed) (3) and in different periods of these interventional endovascular techniques (presented as the publication times), meta-regression was employed. RRs, using variable rates as the dependent variable, and the different interventions, the different health conditions, and the publication times as the independent variables, were determined. There was no evidence that the different interventions, the different health conditions and the publication times were confounding factors in this subgroup analysis. Data from the analyses of moderator variables are presented in Table 3.

**Table 3:** Univariate meta-regression for restenosis

Trial feature	Classification	Number of trials	Beta coefficient	95% CI	P value
Dichotomous outcomes					
Intervention	PTA or Stent	PTA: 2 Stent: 18	0.262	(-0.23, 0.76)	0.281
Smoking	Proportion of the population	≥ 30%: 7 < 30%: 12	-0.113	(-0.49, 0.26)	0.540
History of hypertension	Proportion of the population	≥ 50%: 16 < 50%: 4	-0.004	(-0.38, 0.37)	0.982
History of hyperlipemia	Proportion of the population	≥ 50%: 16 < 50%: 3	0.029	(-0.40, 0.46)	0.890
Publication time	Date of publication of each study	After 2010: 16 Before 2010: 4	-0.480	(-0.41, 0.31)	0.781

### Sensitivity analysis

**Fig. 10:**
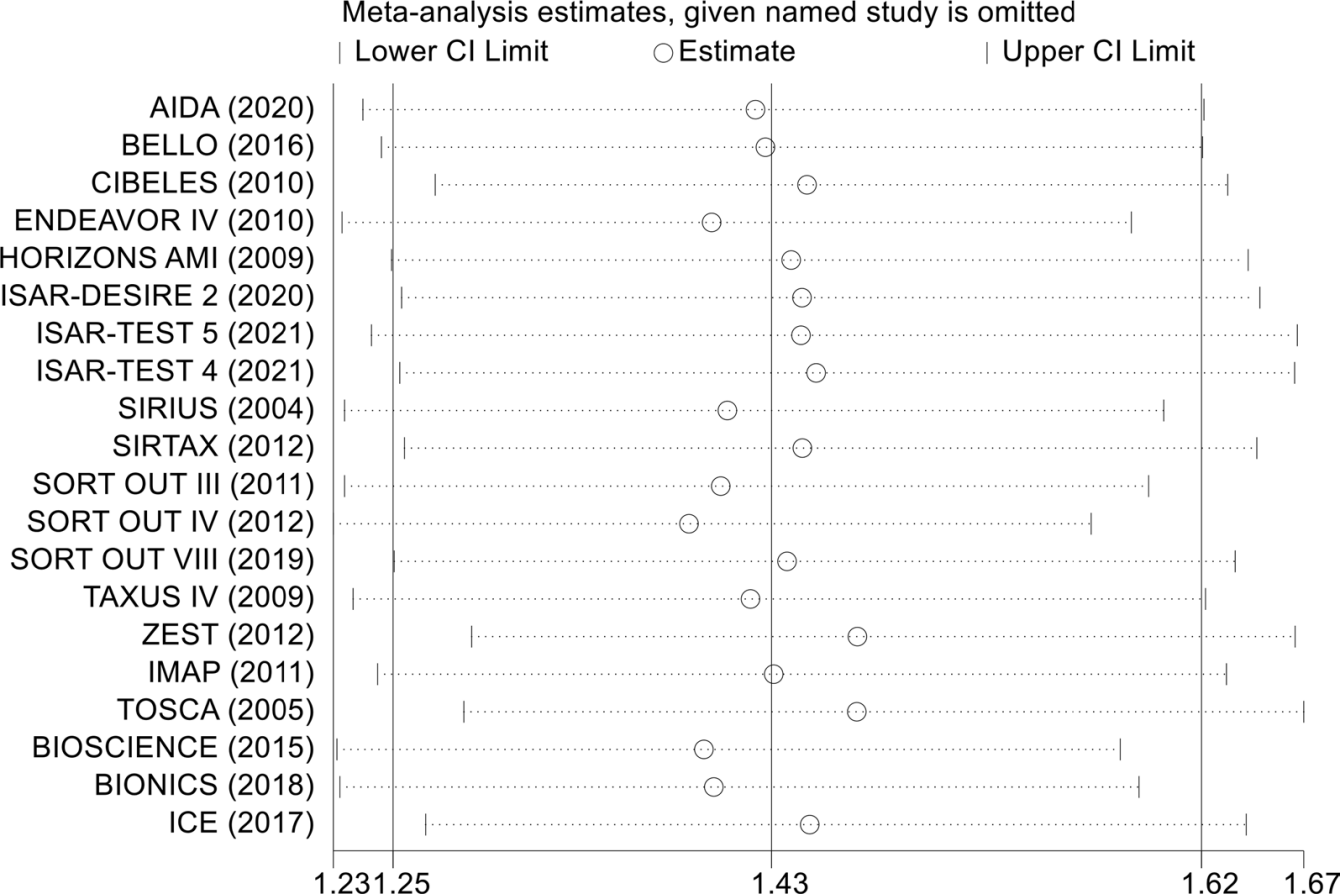
Sensitivity analysis of the association between diabetes mellitus and the endpoints

In the sensitivity analysis, each included study was removed one by one, and a summary analysis of the remaining studies was performed to assess whether a single included study had an excessive impact on the results of the entire meta-analysis (Fig. 10). None of the studies had an excessive impact on the results of the meta-analysis, indicating that the results of the meta-analysis were stable and reliable.

### Publication bias

**Fig. 11:**
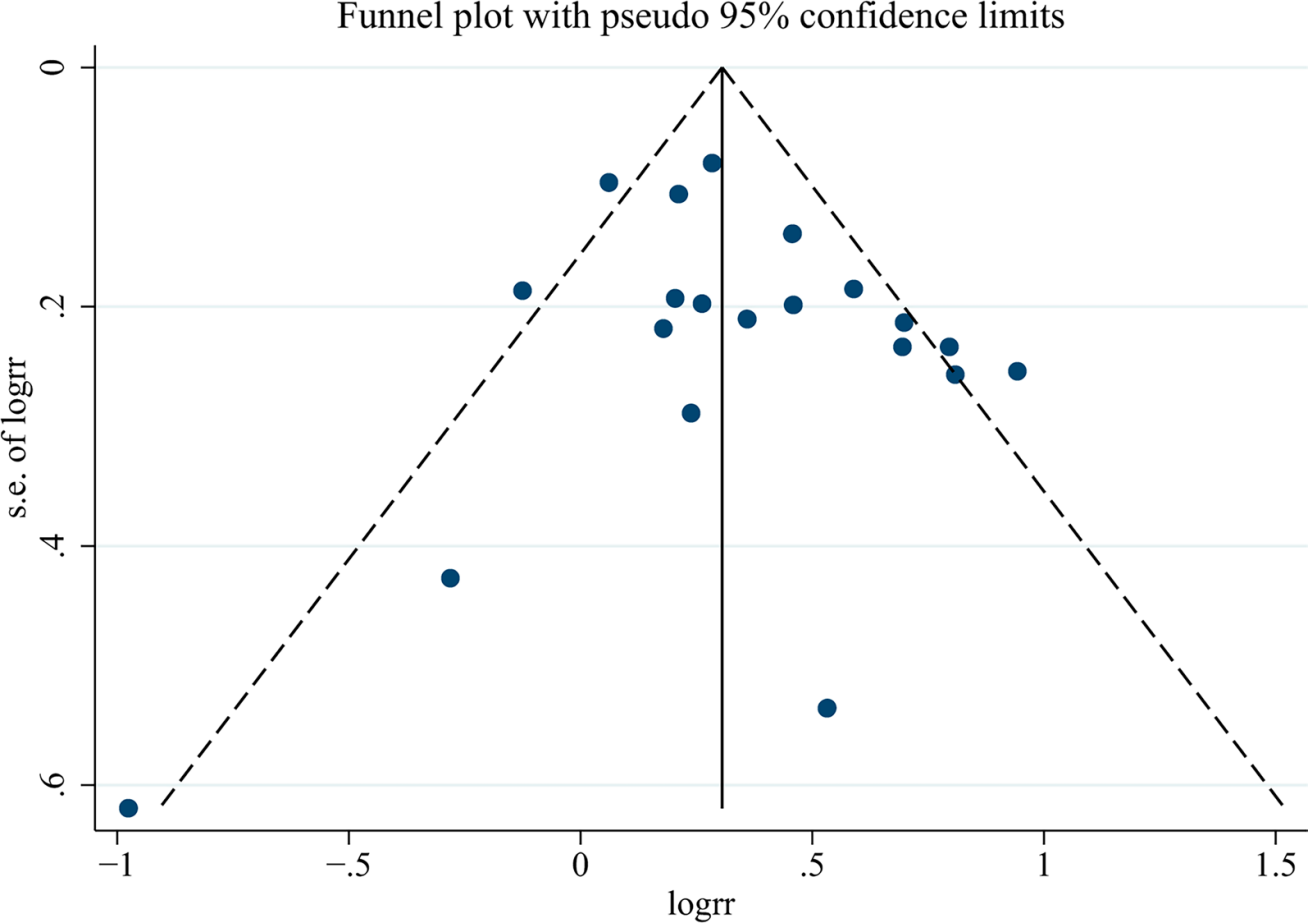
Funnel plot for assessing publication bias

The probability of publication bias in the spread of the meta-analysis by funnel diagram and Begg’s test at a significance level of 0.05 indicated no bias of spread in the present study (p = 0.344) (Fig. 11). According to the results of the diagram, the publication offset of the included studies was small, and the results of the meta-analysis had high uniformity.

### Meta-analysis GRADE assessment

**Fig. 12:**
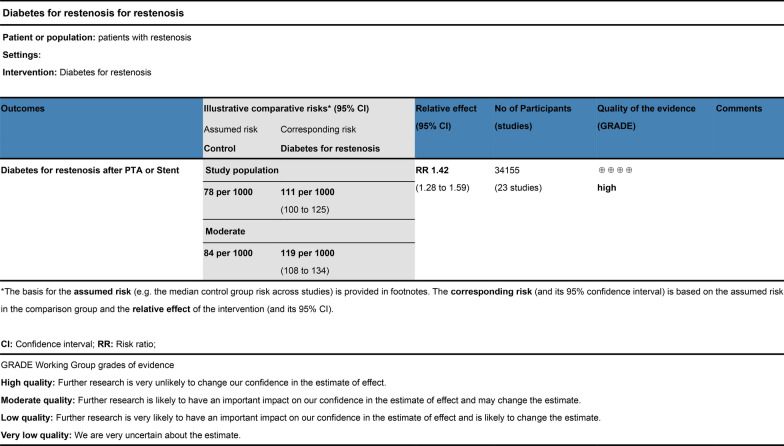
Meta-analysis GRADE assessment. Search strategy of PubMed/Medline. PubMed platform. Searched from 1990 to December 12, 2022. #1 (((diabetes mellitus) AND percutaneous transluminal angioplasty)) OR ((diabetes mellitus) AND stent) (798). #2 ((Percutaneous Transluminal Angioplasty) OR (Transluminal Angioplasty) OR (Endoluminal Repair) OR (Angioplasty) OR (Stent) OR (Endovascular Stent Grafting) OR (Stent Grafting) OR (Stents)) AND ((Diabetes mellitus) OR (Diabetes) OR (Diabetic)) AND ((Restenosis) OR (Graft Restenosis) OR (Restenoses)) AND ((random) OR (randomized) OR (randomised)) (235). #3 ((((diabetes mellitus) OR (diabetes)) OR (melituria)) OR (diabetic)) AND (restenosis) (316)

The evidence was assessed according to the GRADE process for the purposes of making clinical practice recommendations. We used GRADE to evaluate the quality of evidence, as shown in Fig. 12. Judgments about evidence quality (high, moderate, low or very low) were made by two review authors who worked independently and resolved disagreements by discussion. Conclusions were justified, documented, and incorporated into the reporting of results for each outcome. A ‘high’ level of evidence score was obtained according to the GRADE scoring rule after assessing the risks of inconsistency, indirectness, imprecision and publication bias.

## Discussion

At present, PTA or stent implantation is the main treatment for cardiovascular stenosis or occlusion; however, restenosis after endovascular treatment is still a challenge [20]. Indeed, as the number of stent placements has risen to an estimate of over 3 million annually worldwide, revascularization procedures have become much more common [21]. However, restenosis after endovascular therapy is a major problem, and studies have shown that 30% to 50% of patients with coronary ischemic disease experience restenosis after endovascular therapy. To date, DM has been recognized as a high-risk factor for cardiovascular events [7, 22]. Epidemiological investigations have shown that patients with concomitant DM and PAD are at high risk for major complications, such as amputation [23]. Technical progress, such as the application of drug-coated balloons or drug-eluting stents [20, 24], has been found to potentially increase patency after endovascular treatment and thus reduce restenosis. The studies we enrolled included a large number of RCTs that involved drug-coated balloons and drug-eluting stents. Although the incidence of restenosis was significantly lower than that of traditional balloons or bare-metal stents, restenosis remained at ahigh rate and was difficult to resolve.

A study published in 1999 found that the long-term need for TLR increased with higher classes of in-stent restenosis (ISR) (hazard ratio (HR) = 1.7; P = 0.0380) and with the presence of diabetes (HR = 2.8; P = 0.0003) [25]. Taken together with other evidence, DM is suggested to be a strong determinant of restenosis (neointimal hyperplasia) [26, 27]. Michael Jonas et al. [28] used the insulin resistance model of the Zucker fatty rat and found that insulin-resistant Zucker fatty rats developed a thicker neointima and a narrower lumen area 2 weeks after implantation of an abdominal aortic stent compared with normal Zucker lean rats. Additionally, Manikandan Panchatcharam et al. [29] established a femoral artery guide wire injury model in hyperglycemic mice and confirmed that hyperglycemia had an obvious accelerating effect on intimal regeneration after vascular injury by promoting smooth muscle cell proliferation and migration. These animal studies demonstrated the role of hyperglycemia and ISR in regulating the function of vascular smooth muscle cells (VSMCs) and promoting neointimal hyperplasia. Moreover, advanced glycation end products (AGEs) also play an important role in promoting the progression of diabetic vascular disease. Zhongmin Zhou et al. [30] demonstrated a prominently increased accumulation of AGEs and immunoreactivities of receptor for advanced glycation end products (RAGEs) in response to balloon injury in diabetic compared with nondiabetic rats. Additionally, blockade of RAGE/ligand interaction significantly decreased VSMC proliferation in vitro and bromodeoxyuridine (BrdU)-labeled proliferating VSMCs in vivo, suppressed neointimal formation and increased luminal area in both diabetic and nondiabetic rats. These animal studies demonstrated that the pathophysiological features of diabetes, including ISR, metabolic syndrome, hyperglycemia, and increased AGEs, are involved in neointima after balloon dilation or stent implantation.

The current mainstream view is that DM increases the risk of restenosis [31]. Studies have shown that patients with insulin-dependent DM are at particularly high risk for adverse events after percutaneous coronary intervention (PCI) [27]. The universally accepted hypothesis for this phenomenon is that hyperglycemia induces endothelial dysfunction and a proinflammatory state that promote the production of growth factors and cytokines, leading to extensive neointimal formation and thus contributing to the progression of restenosis [32, 33]. Besides the endothelial cell inflammation hypothesis, researches indicated that endothelial progenitor cells played a significant role in restenosis [34, 35]. Balestrieri ML et al [36] revealed that high glucose concentration decreased the quantity of endothelial progenitor cells via SIRT1 signaling pathway. In addition, the prethrombotic environment of patients with diabetes ultimately increases the risk of restenosis [37]. However, until now, there has been no conclusive, large-scale clinical evidence to support this view. Our meta-analysis involved 20 RCTs from multiple countries and time spans, with up to 31,066 patients. The results of the meta-analysis confirmed for the first time that DM is a high-risk factor for restenosis after endovascular treatment in a human cohort.

Studies [38] have shown that the type of glucose-controlling drug can affect postoperative restenosis in diabetic patients after coronary stent or balloon dilation. Their data, especially regarding metformin and thiazolidinediones, indicate beneficial results compared to insulin and sulfonylurea for restenosis. However, no large trials have been undertaken in which the effect of glucose-lowering agents on restenosis is associated with improved outcomes. Indeed, experts believe that maintaining proper glycemic control is crucial for diabetic patients who have undergone revascularization procedures [21]. In several recent prospective studies, high glycemic levels [39, 40] and insulin resistance [41] have increased restenosis rates after coronary stenting or balloon angioplasty, but these results were based on statistical analysis of clinical phenomena, lacking evidence from high-quality controlled studies. Marfella R et al [42] conducted a RCT involving 165 patients with high blood glycemic and ST-segment elevation myocardial infarction (STEMI) undergoing PCI treatment, randomly assigning these patients to an interventional-glycemic-control group and an intensive-glycemic-control group. The results showed that the restenosis rate of patients in the intensive-glycemic-control group after PCI reduced by half (48% and 24%) at 6 months. The study confirmed the positive correlation between blood glucose levels and restenosis. However, it solely concentrated on blood glucose levels without addressing whether the patients had diabetes or specific subtypes. Our research indicates that restenosis of the diabetic patients after interventional treatment is not directly related to blood glucose level. This implies that diabetes may pose other risks aside from high blood glucose, such as AGEs and insulin resistance, which could potentially contribute to restenosis. Although Mone P et al [43] demonstrated the effect of high glucose on the risk of restenosis in STEMI patients without DM, the restenosis of high glucose-DM group (18.5%) is still higher than high glucose non-DM group (14.0%) at one-year follow-up, which indicates that diabetic patients may be influenced by additional pathogenic factors beyond elevated blood glucose levels. In animal studies, it has been demonstrated that hyperglycemia and insulin resistance lead to intimal hyperplasia after vascular injury in rats [28–30, 44, 45], which seems to be of crucial importance in determining exaggerated neointimal hyperplasia after balloon angioplasty in diabetic animals. However, our meta-analysis showed that blood glucose levels in diabetic patients did not affect the incidence of restenosis after endovascular therapy (Fig. 5). In animal experiments, hyperglycemia can contribute to neointimal hyperplasia, which was inconsistent with the results of our meta-analysis, suggesting that there may be other important risk factors involved in restenosis in diabetic patients. A prospective observational study involving 377 participants discovered that postoperative restenosis rates differed among patients with type 2 diabetes and acute myocardial infarction (AMI) based on whether they were prescribed oral sodium/glucose cotransporter 2 (SGLT2) inhibitors [46]. The study confirmed that the administration of SGLT2 inhibitors to type 2 diabetes patients was associated with a reduced frequency of ISR-related events, independent of glycemic control. This research highlights the importance of non-glycemic factors in the reduction of postoperative restenosis among diabetes patients. AGEs and their receptor isoforms seem to have an important contribution to both the pathogenesis and clinical outcome of restenosis. AGEs have been shown to promote carotid intimal regeneration in rats after balloon injury [30], suggesting that AGEs may act as a stimulus for restenosis. Cristiano Spadaccio et al. found that soluble RAGE (sRAGE) levels and total circulating AGEs were positively correlated with an increased risk of stent restenosis [47, 48]. This suggests that AGEs may play a more important role in restenosis than hyperglycemia. Currently, there is a lack of clinical studies on the relationship between restenosis and AGE levels, and further RCT studies may be of great significance.

DAPT has become essential in daily clinical practice. In fact, current practice guidelines recommend aspirin and clopidogrel DAPT for patients suffering from CAD or monotherapy for patients with symptomatic PAD, regardless of clinical background [49]. However, there is controversy over the duration of antiplatelet therapy in view of the different lesion sites and stent types [50]. Cristian A Dámazo-Escobedo et al [51]. confirmed that long-term antiplatelet therapy after coronary stenting would be justified by the high incidence of thrombosis-restenosis through a prospective observational study. However, based on the current European guidelines for management after coronary stent placement, there is no consensus on the ideal duration of DAPT to prevent stent thrombosis-restenosis without a significant increase in bleeding risk. In our meta-analysis, based on the circumstances of antiplatelet therapy included in the study after revascularization, we took 6 months of DAPT as the line of demarcation and found that different durations of antiplatelet therapy had no significant effect on the incidence of restenosis after endovascular therapy. Moreover, anticoagulation therapy after revascularization in PAD is particularly important compared to that after coronary revascularization. The COMPASS and VOYAGER PAD RCTs showed that a low-dose oral anticoagulant combined with aspirin (Rivaroxaban 2.5 mg twice a day; Aspirin 100 mg once a day) improved the long-term patency rate of lower extremity artery disease after endovascular therapy, reduced the incidence of major limb adverse events and cardiovascular events, and did not increase the risk of fatal major bleeding [52–54]. Regrettably, due to the lack of available anticoagulant therapy regimens in the included studies, we did not perform a subgroup meta-analysis of anticoagulant therapy and restenosis in this study. Anticoagulation combined with antiplatelet therapy may be beneficial in future RCTs.

It is worth mentioning that according to the subgroup analysis, patients with diabetes mellitus had a higher TLR rate, while patients with restenosis detected by angiographic follow-up showed no significant difference. This implies that patients with diabetes may be more prone to symptomatic restenosis and require surgical reintervention, suggesting to clinicians that patients with diabetes may have increased reoperation rates. Therefore, diabetic patients should take this characteristic into full consideration when choosing the type of balloon or stent, such as the choice of a drug-eluting balloon to reduce restenosis. Since this study did not involve comparisons of balloon types or stent types, research in this direction may be of great significance for diabetic patients.

The findings of the meta-analysis involving 31,066 individuals affirm that patients with diabetes mellitus (DM) have a higher risk of restenosis following intravascular treatment. Nevertheless, there are still limitations to this meta-analysis. More detailed baseline characteristics of patients were not obtained from some of the included studies, even after contact these corresponding authors, such as the medical history time of DM, detailed blood glucose levels and hypoglycemic methods (such as diet, exercise or drug therapy), which may be important factors and could affect the analysis of restenosis. Due to the high rank of the GRADE results, we also suggest a cautious interpretation for this meta-analysis, and further high-quality RCTs are needed to improve the current conclusion.

## Conclusions

In summary, the findings of this systematic review and meta-analysis provided convincing evidence that patients with DM had an increased risk of primary restenosis after PTA or stenting, suggesting that DM is a high-risk factor for restenosis after endovascular treatment, irrespective of blood glucose level, antiplatelet therapy duration, targeted lesion vessel and continent. In conclusion, our meta-analysis provides a reliable suggestion for the health management of diabetic patients with vascular occlusive disease after endovascular therapy.

## Data Availability

No datasets were generated or analysed during the current study.

## Abbreviations

ABR: Angiographic binary restenosis
AGEs: Advanced glycation end products
BrdU: Bromodeoxyuridine
CAD: Coronary artery disease
CI: Confidence interval
DAPT: Dual antiplatelet therapy
DM: Diabetes mellitus
FU-T: Follow-up time
GRADE: Grading of Recommendations Assessment, Development and Evaluation
HR: Hazard ratio
ISR: In-stent restenosis
MACE(s): Major adverse cardiac event(s)
PAD: Peripheral artery disease
PCI: Percutaneous coronary intervention
PRISMA: Preferred Reporting Items for Systematic Reviews and Meta-Analyses
PTA: Percutaneous transluminal angioplasty
QA-R: Quantitative angiography-confirmed restenosis
RAGEs: Receptor for advanced glycation end products
RCTs: Randomized controlled trials
RR: Relative ratio
SGLT2: Sodium/glucose cotransporter 2
sRAGE: Soluble RAGE
STEMI: ST-segment elevation myocardial infarction
TLR: Targeted lesion revascularization
VSMCs: Vascular smooth muscle cells
